# Immunopathology of Renal Tissue in Fatal Cases of Dengue in Children

**DOI:** 10.3390/pathogens11121543

**Published:** 2022-12-15

**Authors:** Lucca de Lima Siqueira Oliveira, Felipe de Andrade Vieira Alves, Kíssila Rabelo, Leandro Junqueira Moragas, Ronaldo Mohana-Borges, Jorge José de Carvalho, Carlos Basílio-de-Oliveira, Rodrigo Basílio-de-Oliveira, Fernando Colonna Rosman, Natália Gedeão Salomão, Marciano Viana Paes

**Affiliations:** 1 Laboratório Interdisciplinar de Pesquisas Médicas, Instituto Oswaldo Cruz, Fundação Oswaldo Cruz, Rio de Janeiro 21040-900, Brazil; 2 Laboratório de Ultraestrutura e Biologia Tecidual, Universidade do Estado do Rio de Janeiro, Rio de Janeiro 20551-030, Brazil; 3 Laboratório de Genômica Estrutural, Instituto de Biofísica Carlos Chagas Filho, Universidade Federal do Rio de Janeiro, Rio de Janeiro 21941-902, Brazil; 4 Departamento de Anatomia Patológica, Universidade Federal do Estado do Rio de Janeiro, Rio de Janeiro 20270-004, Brazil; 5 Departamento de Anatomia Patológica do Hospital Municipal Jesus, Rio de Janeiro 20550-200, Brazil

**Keywords:** dengue virus, kidney, histopathology, biomarkers, inflammatory mediators

## Abstract

Dengue virus (DENV) infection represents a worldwide public health concern and can cause damage to multiple organs, including the kidney. In this work, we investigated the histopathological changes caused by dengue virus infection along with the detection of inflammatory mediators, cytokines, and cell expression patterns in the renal tissue of three fatal cases in children. Hematoxylin and Eosin staining was performed to analyze these histopathological changes. Immunohistochemistry allowed for the detection of immunological inflammatory markers in renal tissues that were quantified and further analyzed. Vascular congestion, edema and glomerular infiltrate were observed in the three cases, in addition to the thickening of the matrix area around the glomerular capillaries and mononuclear infiltrate associated with vascular congestion in the medullary region. The renal tissues exhibited collagen deposition and high expression of CD68^+^ Mø, CD8^+^ T, CD56^+^ cells and MMP-9, and the cytokine profile was mainly characterized by the expression of IFN-γ and TNF-α. Additionally, the expression of RANTES, VEGFR-2 and VCAM-1 were observed. The replication of DENV was evidenced by the detection of the NS3 protein. These results contributed to clarifying the main factors that may be involved in changes in the renal tissue of fatal cases of dengue in children.

## 1. Introduction

Dengue virus infection is considered to be a global public health challenge in tropical and subtropical countries by the World Health Organization (WHO), given the favorable environmental conditions for the life cycle of *Aedes* mosquitoes in these locations [1]. The disease registered a 30-fold increase in the world between 1960 and 2010, which can be explained by several factors, such as an increase in the population growth rate, inadequate urban planning, exponential growth in the frequency of air travel and precarious health services, in addition to the difficulty in accessing it [1,2,3,4].

Since 2009, the dengue disease classification has included two categories: Non-severe dengue and severe dengue. Non-severe dengue includes two sub-categories, corresponding to cases with warning signs and without warning signs. The use of this nomenclature was adopted to favor patient triage and thus make it possible to detect cases that may evolve to severe dengue, an advanced stage of the disease that is determined by the presence of one or more of the following manifestations: Plasma leakage that maylead to shock (dengue shock) and/or fluid accumulation, with or without respiratorydistress; severe bleeding; and severe organ impairment [1,5].

Dengue virus (DENV) infection, like other arbovirus infections, is associated with dysfunctions in different organs [6,7]. The correlation between dengue virus infection and renal injury have been demonstrated in adults [8,9,10] and in children [11,12]. In this context, the dengue virus infection can cause glomerulonephritis (inflammation of the renal glomerulus), lead the patient to show signs of acute renal injury (ARI) and consequently triggers proteinuria (abnormal presence of proteins in the urine) and hematuria (abnormal presence of erythrocytes in the urine), which usually occur during or shortly after the acute phase of infection [13].

Different studies support the use of the evaluation of alterations in serum levels of creatinine and urea as a parameter to measure the function state of this organ [14,15]. Previous reports of our group observed morphological alterations and detected dengue virus antigens, and pro-inflammatory cytokines/chemokines, such as IFN-γ, TNF-α and RANTES, in the kidney of dengue virus-infected adult patients, as well as the subpopulation of CD4^+^ T, CD8^+^ T and CD68^+^ Mø cells [7,10,16]. Additionally, we also observed a fibrosis process along with the detection of other inflammatory mediators and biomarkers, such as adhesion molecules in renal tissue. These findings could also be fully or partially noted in other studies approaching dengue virus infection consequences in different organs (heart, spleen, thymus, and pancreas) in mice and humans [16,17,18,19].

Therefore, the evaluation of renal tissues from fatal dengue cases in children can emerge as a valuable tool in understanding the role of the development of renal affection in viral infections in children. With this perspective, in this work we analyzed biochemical parameters, histopathological and immunopathogenic aspects caused by dengue in the renal tissue of three children. The analysis of these biomarkers and associated damages mentioned above may help clarify immune response mechanisms involved in the renal pathogenesis of dengue in fatal cases in children. This work addresses cases of the infection and aspects of it in renal tissue in children, a topic that raises questions to be solved, involving epidemics that caused a significant number of deaths in this age group.

## 2. Results

### 2.1. Levels of Serum Creatinine and Urea in the Cases Studied during Hospitalization Days

Levels of creatinine and urea were measured during the hospitalization. Case 1 presented serum levels above the reference values (0.9236 mg/dL and 54.54 mg/dL, respectively) for both substances (Table 1), which could already indicate a possible renal dysfunction. Meanwhile, cases 2 and 3 presented values within the reference levels (0.6 mg/dL and 18.0 mg/dL; 0.5 mg/dL and 19 mg/dL), not indicating, at first, such dysfunction (Table 1). Reference values for creatinine [20] and urea [21] were adopted according to reports in the clinical literature.

### 2.2. Histopathological Analysis

**Figure 1:**
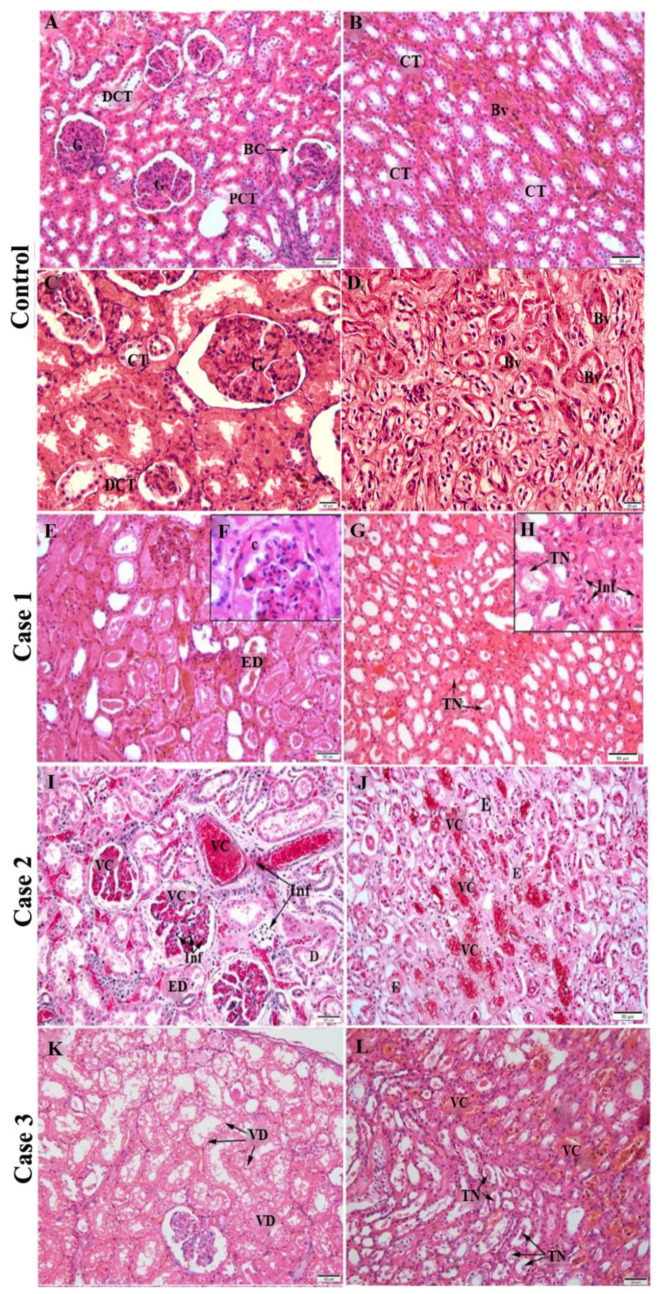
Histopathological analysis of fatal cases of dengue in children compared to control (Hematoxylin and eosin stain): (**A/B**) Renal control tissue showed regular vessels, tubules in the cortical and medullary regions and regular glomeruli in the cortical region. (**C**) 400× magnification of control tissue showed regular glomeruli, distal convoluted and collecting tubules in cortical region and (**D**) regular functional blood vessels in medullary region. (**E**) Renal tissue from fatal case 1 presents epithelial denudation in cortical region in addition to (**F**) the presence of cube-shaped epithelial cells in Bowman’s space in 1000× magnification (1000×) of cortical region. (**G**) Tissue necrosis was observed in the medullary region. (**H**) Tissue necrosis and inflammatory infiltrate was observed in higher magnification (1000×) of medullary region. (**I**) Case 2 tissue evidenced vascular congestion, inflammatory infiltrate presence and dilated proximal tubules in cortical (**J**) and edema in medullary. (**K**) Vacuolar degeneration was observed in renal tissue’s cortical region from Case 3, along with (**L**) vascular and tissue necrosis in the medullary region. Bv—Blood vessels; PCT—Proximal convoluted tubule; DCT—Distal convoluted tubule; CT—Collecting tubules; G—Glomeruli; BC—Bowman’s capsule; VC—Vascular congestion; E—edema; c—growth/proliferation of cube-shaped epithelial cells; ED—Epithelial denudation; Inf—Inflammatory infiltrate; VD—Vacuolar degeneration; TN—tissue necrosis; D—Tubular dilation.

In renal histology, when analyzing the cortical region, it is possible to observe the following structures: Renal Glomeruli, distal convoluted tubule, proximal convoluted tubule and Bowman’s capsule (Figure 1A), while in the medullary region, collecting tubules and blood vessels are evident, as they are present in both histological regions (Figure 1B) [22]. Regular structures of renal tissue are also evident in higher magnifications of control tissue, providing an interesting contrast with other cases, as glomeruli, distal convoluted and collecting tubules all have expected healthy morphology in the cortical region (Figure 1C) as well as blood vessels in the medullary region (Figure 1D).

The histopathological analysis showed no changes in the control tissues of parenchyma (Figure 1A–D). As expected, all the component structures of the renal cortical and medullary regions were regular and well preserved. The evaluation of the renal tissue cortical region of the fatal cases studied showed alterations, such as proliferation of the parietal epithelium of the Bowman’s capsule (Figure 1F), epithelial denudation in groups of proximal tubules (Figure 1E,I), as well as the presence of diffuse areas of vascular congestion in the capillary loops (Figure 1I), inflammatory infiltrate (Figure 1I), dilation of contorted tubules (Figure 1I) and foci of vacuolar degeneration (Figure 1K). The analysis of the renal medullary region of these fatal cases evidenced the existence of foci of tissue necrosis (Figure 1G,L), influx of inflammatory cells (Figure 1H) and intratubular edema (Figure 1J), in addition to congestion of peritubular capillary loops (Figure 1J).

**Figure 2:**
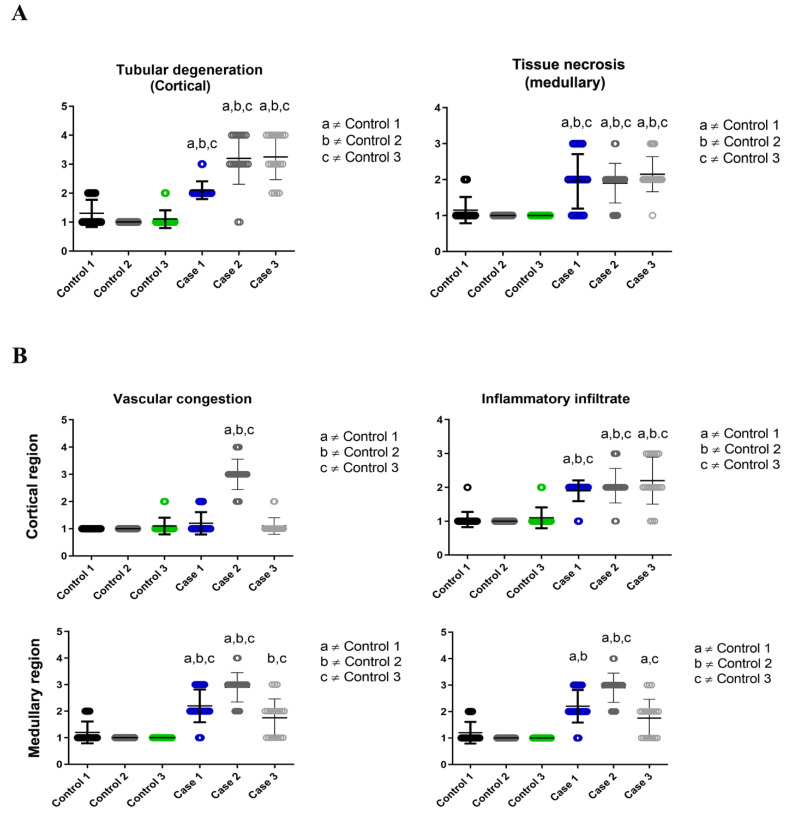
Quantifications of damage observed in cortical and medullary region of the kidney of the cases studied: (**A**) Cases 2 and 3 presented intense degeneration in their tubules. (**B**) The blood vessels of the renal tissue tended to present congestion more frequently in case 2 and the presence of inflammatory infiltrate occurred with similar expressiveness in the cortical region of the cases studied, while it was more quantitative in the medullary region of cases 2 and 3. Controls 1, 2 and 3 showed no signs of relevant tissue damage. The number used for both controls and cases was 3 (n = 3). Controls 1-3 and cases 1-3 each represent a single and different patient. Mann-Whitney test and t-test was used. Data represent the mean ± SDM. Statistically significant differences (*p* < 0.05) between each fatal dengue case and each control (a,b,c). The cases revealed a significant increase in the degree of damage compared to controls in most of the comparisons.

In order to better systematize the findings in the renal tissue, the quantification was carried out according to the following parameters: Tubular degeneration, tissue necrosis, vascular congestion and presence of inflammatory infiltrate. Severity of tissue damage was classified in five levels (0 = none; 1 = low; 2 = moderate; 3 = severe; 4 = very severe). These quantification criteria were adopted according to a previous study that sought to measure the level of organ (pancreas) damage in fatal cases of dengue in adults, based in histopathological alterations [17]. All parameters were observed in the majority of the 40 fields captured for each case and in less than 10 control fields. The criteria of tubular degeneration had a severe level of involvement (3/4 quadrants of the field) in most of the cases’ fields (Figure 2A) while the other parameters of damage adopted were expressed in a slightly different pattern between cases, mostly being low or medium (Figure 2A,B).

### 2.3. Evaluation of the Fibrosis by Collagen Quantification and MMP-9 Detection in Renal Tissues

**Figure 3:**
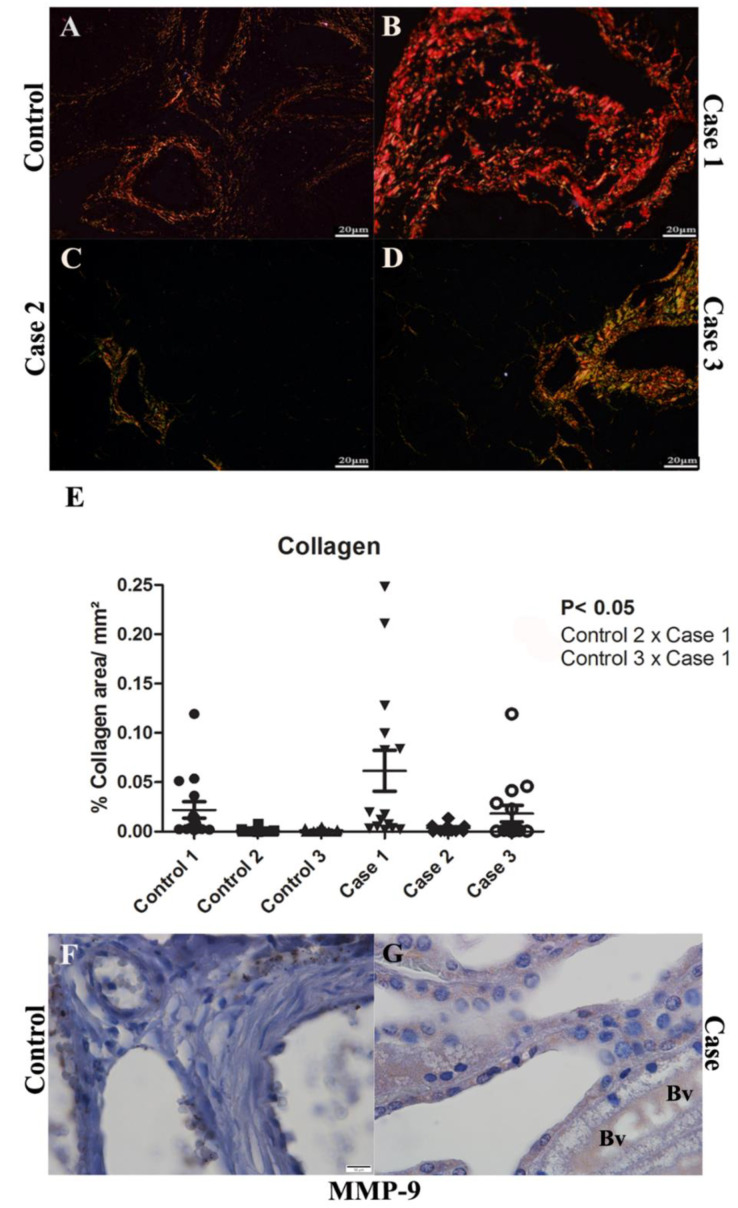
Analysis of tissue collagen in the kidney by PicroSirius Red stain and MMP-9 detection: (**A**) Control renal tissue exhibited regular distribution around vessels, tubules and glomeruli in the cortical region; distribution of tissue collagen in the cortical region of renal tissue in cases (**B**) 1, (**C**) 2 and (**D**) 3, respectively. (**E**) The quantification of the percentage of collagen/mm^2^ was performed in the three controls and three cases. Case 1 showed a significant increase in relation to controls 2 and 3. (**F**) Control tissue did not show significant detection of MMP-9. (**G**) Expression of MMP-9 in the blood arterioles in the medullary region. Bv—Blood vessels; Mo—Monocytes. (**A**–**D**): Picro Sirius Red stain of cortical region; (**F**–**G**): Immunohistochemistry technique.

In order to analyze fibrosis in renal tissues, Picro Sirius Red staining was performed as it is known to evidence collagen deposition. Control tissues (Figure 3A) exhibited normal collagen deposition, present around vessels, with regular thickness. It was possible to observe an extensive area of collagen deposition around the arched vessels in the cortical region of case 1 (Figure 3B), as that was not evident in the other two cases studied (Figure 3C,D). The quantification analysis only showed a significant difference in the collagen deposition in the renal tissue of case 1, compared to the controls, indicating the fibrogenic pathway activation as a possible action mechanism of DENV in this tissue (Figure 3E). Corroborating and helping in the interpretation of these findings, Metalloproteinase-9 (MMP-9), an enzyme that participates in the degradation of the extracellular matrix, was detected in blood vessels in the medullary region (Figure 3G).

### 2.4. Detection of Viral Antigen in Infected Renal Tissue

**Figure 4:**
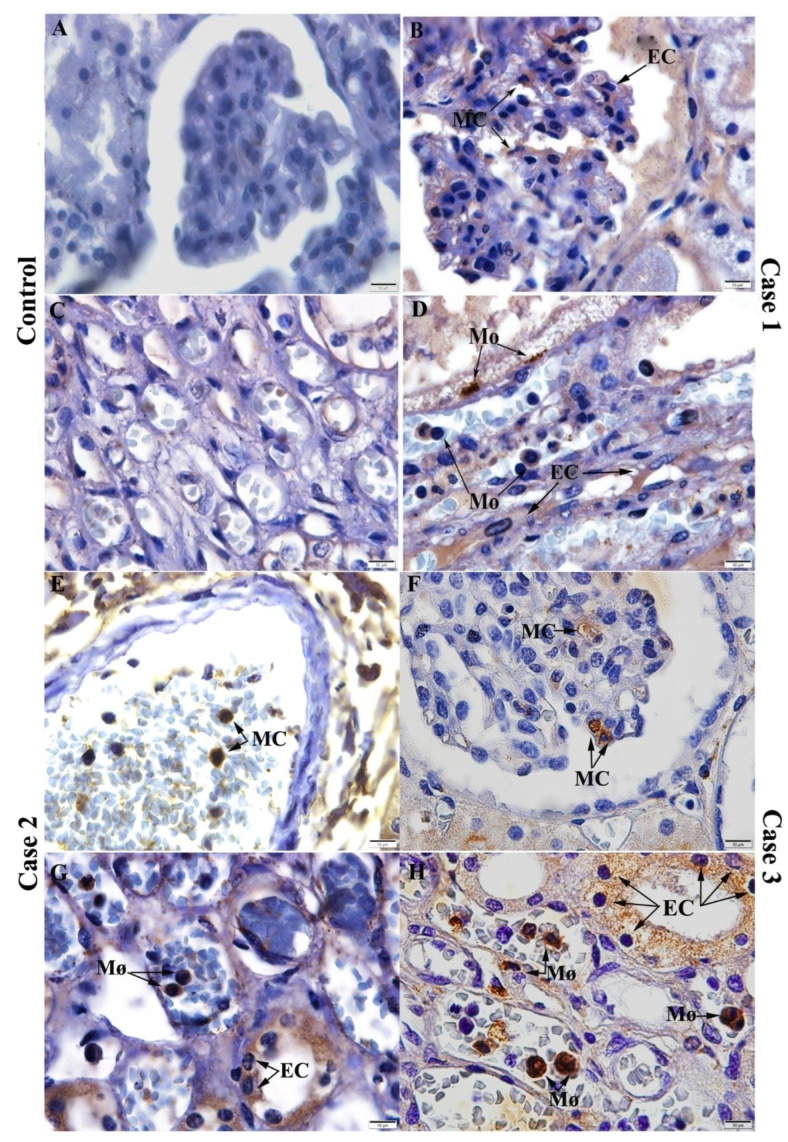
Detection of the NS3 protein antigen in the renal tissue: (**A**,**C**) Control renal tissue showed no expression of the NS3 protein. Detection of NS3 protein in: (**B**) Mesangial and endothelial cells in the cortical region in case 1. (**E**,**F**) Mesangial cells in the cortical region in cases 2 and 3. (**D**,**G**) Endothelial cells and monocytes/macrophages in the medullary region in cases 1 and 2. (**H**) Macrophages and endothelial cells in the medullary region in case 3. MC—Mesangial cells; EC-Endothelial cells; Mo/Mø—Monocytes/Macrophages.

In order to investigate the viral replication in the renal tissue of the cases studied, we performed the detection of NS3 antigen (dengue virus non-structural 3 protein). This protein was detected in the mesangial cells of the cortical region in all three cases (Figure 4B,E,F), as well as in endothelial cells and monocytes/macrophages in the medullary region (Figure 4D,G,H), evidencing viral replication in these cells. Images of the three cases were used for comparison with the control tissue, which, as expected, showed no expression of this antigen (Figure 4A,C).

### 2.5. Characterization of the Cell Expression Pattern in Infected Renal Tissue

**Figure 5:**
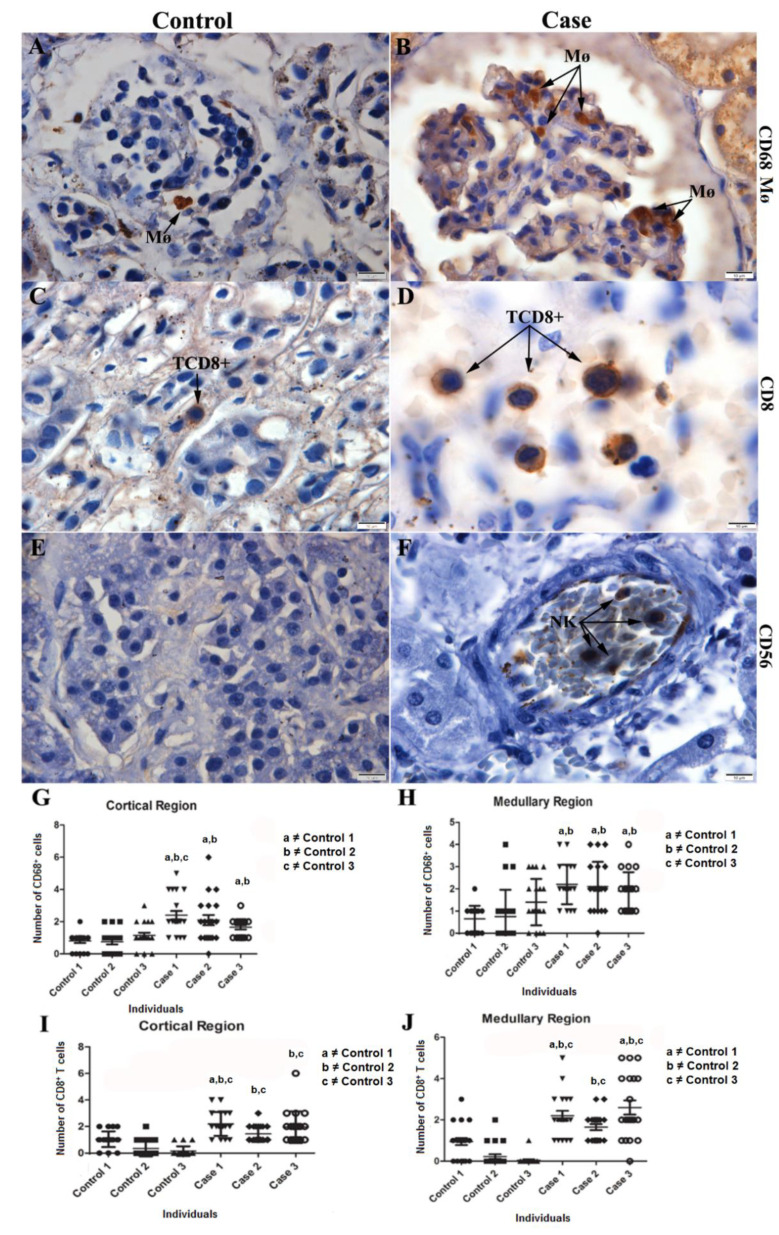
Characterization of cellular subpopulations in the renal tissue and quantification: (**B**) Detection of macrophages (CD68^+^ Mø cells) in the renal tissue of Case 3 in the cortical region. (**D**) Renal tissue from a Case 3 with expression of CD8^+^ T cells in the medullary region. (**F**) Expression of CD56^+^ cells in circulating NK cells in the medullary region in Case 2. (**A**,**C**,**E**) Control tissue with little or no expression of the markers used. (**G**,**H**) Quantification of CD68^+^ Mø cells. (**I**,**J**) Quantification of CD8^+^ T cells. Mø—Macrophage; TCD8^+^—CD8^+^ T lymphocyte; NK—Natural Killer Cells. For the statistical analysis, n = 3 was used for controls and cases. Controls 1–3 and cases 1–3 each represent a single and different patient. Mann-Whitney test and t-test was used. Data are represented as mean ± SDM. The letters a, b and c indicate differences that are statistically significant between individual specimens (*p* < 0.05) Mø—Macrophage; TCD8^+^—CD8^+^ T lymphocyte; NK—Natural Killer Cells.

The infiltrates observed in the renal tissue were characterized by the expression of CD68^+^ Mø cells (Figure 5B), showing the presence of macrophages with the characteristic morphology of hyperplasic cells. The detection of CD8^+^ T cells evidenced the presence of cytotoxic T lymphocytes (Figure 5D). In addition, it was possible to detect CD56^+^, pointing to the expression of NK (Natural Killer) cells (Figure 5F). A representative image of each case was selected for the three cases studied (CD68^+^ Mø—Case 3; CD8^+^—Case 3; CD56—Case 2). Such changes were not observed in the control renal tissue (Figure 5A,C,E). Quantification of CD68^+^ Mø positive cells showed a significant increase between cases and controls (Figure 5G,H). CD8^+^ T lymphocytes quantification was also performed, pointing to a statistically significant increase in cases 1 and 3 relative to the controls in both cortical and medullary regions (Figure 5I,J).

### 2.6. Detection of Cytokines, Chemokines and Inflammatory Mediators in Kidney Tissue

**Figure 6:**
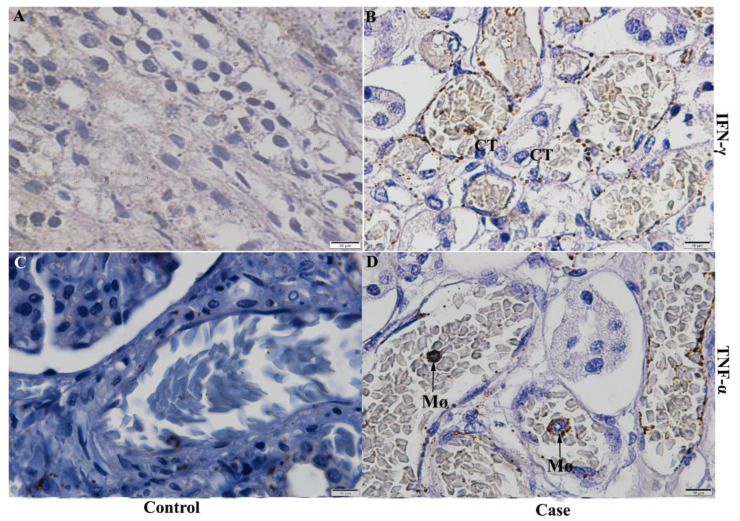
Detection of cytokines in the kidney of fatal cases. (**B**) Detection of IFN-γ expression around and across collecting tubules in medullary region in Case 1. (**D**) Detection of TNF-α expression in circulating macrophages in the medullary region in Case 1. (**A**,**C**) Control tissue did not show significant detection of these markers. CT—Collecting tubules; MC—Mesangial cells; Mø—Macrophage; Mo—Monocytes EC—Endothelial cells.

The pro-inflammatory profile was mainly characterized by the expression of IFN-γ and TNF-α, two classic cytokines involved in dengue virus infection in children and in the progression of its severity. IFN-γ expression was detected around collecting tubules in medullary region (Figure 6B), while TNF-α was expressed by macrophages in the cortical region (Figure 6D). These renal alterations were not significantly observed in the control renal tissue (Figure 6A,C).

**Figure 7:**
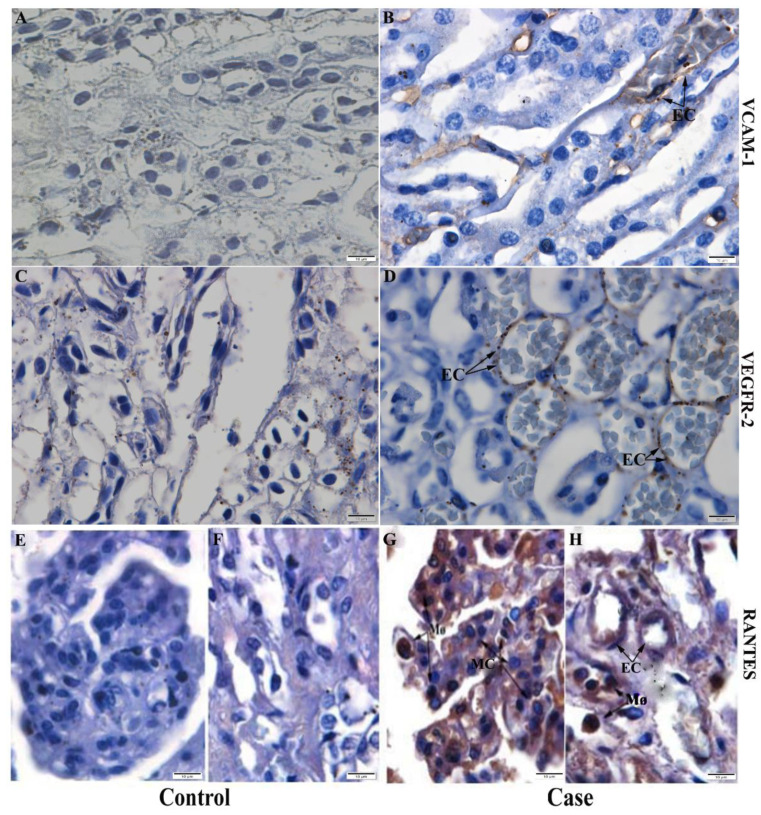
Detection of inflammatory mediators and chemokine in the kidney of fatal cases. (**B**) VCAM-1 expression in endothelial cells in the medullary region in Case 3. (**D**) VEGFR-2 in blood capillary endothelial cells in the medullary region in Case 2. (**G**) Expression of RANTES in mesangial cells and macrophages in the cortical region in Case 1 (**H**) RANTES expression in endothelial cells and macrophages in the medullary region in Case 1. (**A**,**C**,**E**,**F**) Control tissue did not show significant detection of these markers. EC—Endothelial cells; MC—Mesangial cells; Mø—Macrophage.

In addition, it was possible to detect the expression of VCAM-1, an important cell adhesion molecule (Figure 7F) and of inflammatory mediator VEGFR-2, both by medullary region’s endothelial cells (Figure 7B), while chemokine RANTES, also known as CCL5, related to the increase of vascular permeability, was expressed by macrophages and mesangial cells in the cortical region (Figure 7G) and macrophages and endothelial cells in the medullary region (Figure 7H). Such changes were not detected in control tissues (Figure 7A,C).

## 3. Discussion

Dengue, in general, is a disease that has a fast resolution (five to seven days) after the symptoms onset [1,5]. However, there are individuals in whom the disease progresses to a more severe form, with impairment of some organs, including the kidney, a fact that is possibly explained by the occurrence of an inflammatory condition, which leads to tissue injuries and dysfunction [1,5,16,17]

Initially, we analyzed the data of serum creatinine and urea levels in the patients’ charts, as they both are end products of nitrogenous metabolism [23], in order to assess the renal function of the three fatal cases. Creatinine is widely known as a metabolite of muscle creatine catabolism [23,24] and urea is the primary product from dietary protein and tissue protein degradation and synthesis [23,25]. They may reflect the glomerular filtration rate on several occasions and can thus be found at low serum levels in healthy individuals [14,15]. High serum levels of urea and creatinine may indicate a low renal glomerular filtration rate, and consequently, a greater risk of renal injury progression [26]. Our data showed an increase in serum levels of these markers of renal function in case 1. This context suggests that the dengue virus infection could have affected more severely the renal tissue in this case, considering the possibility of the renal failure having a relation with the outcome of the infection, a situation observed in other studies involving the DENV-infected adult patients [27,28]. It is important to highlight that the patient in case 1 also had a positive blood culture test for the bacteria *Pseudomonas aeruginosa* on the third day of hospitalization and this could have played a role in determining the outcome of the case. Secondary bacterial infection in dengue, although rare, can happen and due to the fragility of the patient’s condition, can lead to a serious systemic infection, capable of affecting the cardiorespiratory system [29,30,31,32]. A previous clinical study addressing viral and bacterial co-infection highlighted the hypothesis that the pre-existence of a comorbidity may contribute to the worsening of the condition in these cases, which becomes even more critical with the establishment of the infection [33], evidencing that the differential diagnosis is essential to exclude this possibility.

Previous investigations by our group, involving fatal cases of dengue in adults, showed intense degeneration in the glomerulus region and in the proximal and distal convoluted tubules, as well as in areas of mononuclear cell infiltrate, hemorrhage and perivascular edema in the medullary region [34]. These findings are extremely useful for comparison purposes with the results obtained in the present study, which also focuses on renal tissue infected by the dengue virus but addresses cases that occurred in children. In the histopathological findings of the present study, it was possible to observe alterations such as vascular congestion, tissue necrosis and inflammatory infiltrate, suggesting the impairment of the renal tissue caused by dengue virus infection in children. In case 1, epithelial denudation was observed in the convoluted tubules along with a process of growth/proliferation of the parietal epithelium of Bowman’s capsule, which causes a reduction in the space between Bowman’s capsule and glomerulus. This distance alteration represents an initial indication of glomerular injury [35] and can be related to the increase in serum levels of creatinine and urea in the patient, characteristics that are also associated with the risk of progression of the injury severity to the organ [15] and indicates an acute glomerular infection (renal glomerulitis) that may play a relevant role in the outcome of the infection. In case 2, we could observe numerous foci of vascular congestion in the blood vessels, a trait that, by being responsible for poor renal perfusion and impaired renal function, could be present in a conjuncture of renal ischemia [36]. Ectasia (dilation) and degeneration of the convoluted tubules were also observed, pointing to the possibility of a solute transport-related renal organ dysfunction, as these structures play a fundamental part in the active transport and reabsorption of the majority of solutes in the glomerular filtrate [37]. Case 3, on the other hand, presented foci of vacuolar degeneration, which is an initial indication of most forms of cell damage [38]. This change was observed in the renal tissue of adult patients infected with other viral species, such as SARS-CoV-2 [39], and in children with primary nephrotic syndrome [40]. These previous findings corroborate the idea of organ damage in the present study. Another parameter that was also evaluated, based on quantification, was the presence of inflammatory infiltrate, which represents a process that occurs due to the recruitment of immune cells to the site of infection, which was also observed in previous studies [10,18]. In this study, it was observed in all three cases with similar intensities.

We also performed the analysis of collagen deposition areas in the renal tissue, which may be related to a process of extracellular matrix degradation, which was previously reported in adult patients infected with dengue virus [41,42]. This degradation usually occurs due to the action of proteolytic enzymes called metalloproteinases, that, by changing the composition of the matrix, can encourage the deposition of components such as collagen in adjacent structures, a process that is reported in the early stages of interstitial fibrosis in cases of obstructive nephropathy [43]. It was possible to notice a more intense tissue collagen deposit in case 1, as compared to the other two cases (with less expressive collagen areas). Furthermore, the observation of focal areas of collagen is present in case 1 and even the absence of expressive focal areas in cases 2 and 3 configured an indication of infection markers to be researched during the work, such as MMP-9 (Metalloproteinase-9). MMP-9, detected in blood arterioles in the medullary region of the cases’ samples, is an enzyme with extracellular matrix degradation function [44]. This protein also appears to be involved in increasing vascular permeability and severity of infection [45]. Another recent study indicated that, in dengue virus infection, MMP-9 can also act directly on endothelial cells, hindering cell adhesion and impairing the occlusion junctions of these cells, causing a situation of hyperpermeability observed in severe cases of dengue [46].

Then, using the immunohistochemistry technique, we detected the NS3 protein, an important viral antigen that is indicative of viral replication [47,48]. This protein was detected in mesangial cells in the cortical region and in endothelial cells, monocytes/macrophages in the medullary region of the analyzed cases. Thus, it is possible to state that, after infection, the virus was able to infect and replicate in the renal tissue, leading to the appearance of histopathological changes, directly or indirectly. Dengue viral antigens have also been detected in the medullary region of adult renal tissue in inflammatory cells [49] and in macrophages in the peritubular and perivascular space [10,31].

It was also possible to observe the expression of CD68^+^ Mø cells, showing the presence of macrophages with characteristic morphology of hyperplasic cells. Macrophages are considered one of the main targets of DENV replication in several organs (kidney, heart, thymus, and gastrointestinal tract) of mice and humans [7,18,31]. In addition, CD8^+^ T cells were also detected, with a statistically significant increase in cases 1 and 3, compared to control tissue. Our results suggest that the cytotoxic response could be one of the main mechanisms used in the attempt to control the infection, having a huge role in the modulation of the body’s reaction to the virus. Evidence suggests that cytotoxic T cells may be sufficient to cause the occurrence of severe signs of dengue, in addition to stimulating the expression of inflammatory cytokines, such as Tumor Necrosis Factor (TNF) [50]. CD8^+^ T cells also seem to have effector function in the response to the infection as DENV-specific CD8^+^ T cells can be detectable in the skin of DENV-infected adult patients in the acute stage, suggesting their migration to the site of the infection and their potential to mediate local response [51,52]. Previous studies by the group involving fatal cases of dengue in adults also observed an increase in this cell subpopulation in renal tissue [16]. There are reports of detection of this cell subtype in cases of glomerulonephritis in adult humans, suggesting that T cell-mediated cytotoxicity is an effective mechanism of injury to renal tissue [53]. The detection of CD56^+^ cells (Natural Killer—NK) in the analyzed cases indicates the activation of innate immune response, since NK cells are able to recognize complexes formed between antibodies and virus antigens expressed on the surface of cells infected and initiate the response against them [54].

In severe dengue, although plasma extravasation usually occurs at the end of the acute phase, pathological and physiological reactions begin in the first moments of infection, with the production of inflammatory cytokines by cells exposed to the virus [55]. Several signs related to the kidney during dengue virus infection have already been identified, such as proteinuria, glomerulonephritis and tubular necrosis [56], however the role of this organ in the persistence of the infection is not yet fully elucidated [57].

Thus, in the present work, the profile of inflammatory cytokines was characterized by the expression of IFN-γ, produced by lymphocytes and which activates other cell types, such as NK cells, other lymphocytes [58], and TNF-α, produced mostly by macrophages, which corroborates the findings of previous studies of dengue in renal tissue [10]. Both cytokines can work in the inhibition of viral replication through cell-mediated cytotoxicity [59]. Increased levels of this cytokines have been reported in other organs, such as the liver, in studies involving fatal cases of this arbovirus in children [60] and both of them are able to induce the expression of other markers during an inflammatory state [61]. TNF-α regulates and induces the expression of VCAM-1, a vascular cell adhesion molecule that has a huge role in trans endothelial migration and recruitment of leukocytes during the inflammatory process [62,63]. In this study, it was expressed by endothelial cells, as it may play a role in modifying components of the extracellular matrix in this cell type, inducing an increased plasma leakage [64].

Furthermore, it was possible to observe the expression of the inflammatory mediator VEGFR-2 (vascular endothelial growth factor receptor 2) in the endothelial cells of blood capillaries in the medullary region. VEGFR-2 and its factor (VEGF) are linked to increased vascular permeability and inflammation caused by dengue virus infection in adults [65], stimulating the proliferation and migration of endothelial cells in this process [66]. VEGF regulation is modulated by RANTES, a chemokine that is heavily involved in altering vascular permeability [67]. In the studied cases, the chemokine RANTES was detected in mesangial cells in the cortical region, in endothelial cells from the medullary region and in macrophages from both regions. Previous studies suggest that conformational changes in mesangial cells may stimulate cytokine production in glomerular injuries [68]. This chemokine also seems to be related to the process of angiogenesis, preceded and/or accompanied by vascular permeability in other organs, such as the heart [67,69], a situation that can result in the leakage of plasma, a classic characteristic of severe infections by the virus.

## 4. Materials and Methods

### 4.1. Ethical Procedures

All procedures performed were approved by the Ethics Committee of the Oswaldo Cruz/Fiocruz Foundation, under the CAEE approval number: 47525115.3.3001.5279.

### 4.2. Children Fatal Cases

The renal tissue samples analyzed in this study were obtained from three fatal dengue cases in children that occurred during the Rio de Janeiro dengue epidemics between 2008 and 2012. Samples of renal tissue in paraffin blocks and the medical records were provided by the Pathological Anatomy Department of Hospital Jesus, under the responsibility of Dr. Fernando Colonna Rosman.

### 4.3. Case Presentation

- Controls: Three children who were approximately the same age of the cases (8 to 13 years old) and died of natural causes or trauma, without any history of dengue and other infectious diseases, and without any pathology directly or indirectly related to the kidney.
- Case 1: Male, melanodermic, 7 years and 8 months old, born in Rio de Janeiro, Brazil. Admitted to HMJ (Hospital Municipal Jesus) on 30 January 2008. On 23 January 2008, after five days of high fever, nausea and vomiting, the mother sought medical attention at a health center, being medicated with amoxicillin for diagnosis of “infection”. Not improving, he returned to the health care in serious condition, where the presumptive diagnosis of dengue was made by serology (antibody anti-IgM) and hospitalization was provided. Blood culture was positive for bacterial comorbidity (*Pseudomonas aeruginosa*) after two days of hospitalization, probably acquired in the hospital setting. After 6 days, acute renal failure was diagnosed and requested hemodialysis. After 12 days in hospital, he died on 2 November 2008 and submitted to necropsy. The last blood count performed showed leukocytosis, neutrophilia, lymphopenia, hyperchromic microcytic anemia, and thrombocytopenia. The main diagnosis was hemorrhagic dengue, and the cause of death was extensive bilateral pulmonary hemorrhage. In the analysis of the kidneys proximal tubular was altered due to hydro-electrolyte disturbance, and congestion was present.
- Case 2: Female, 9 years, 11 months, 28 days, born in Itaboraí, Rio de Janeiro, Brazil. On 23 April 2011 started to present fever and general malaise, being taken to the Health Center on 24 April 2011. During physical examination, she presented drowsiness and prostration, but cooperated with the examination. She was also dehydrated, pale, eupneic (respiratory rate of 22 irpm, 20 irpm, 24 irpm), cyanotic, tachycardic (heart rate of 130bpm), with fever and had blood pressure of 90 × 50 mmHg. Petechiae were observed on the face and lower limbs. She required replacement and maintenance hydration with saline and glucose solutions, with the addition of chloride of sodium and potassium chloride. She was transferred to the intensive care unit at a hospital on 25 April 2011 for 10 h and 40 min. In the blood count was noted leukopenia, neutropenia, lymphopenia, anemia, thrombocytopenia. Heart rate reached 160bpm and unmeasurable blood pressure (cold shock). Despite the efforts to maintain cardiovascular and respiratory functions, she did not resist. There was a drop in heart rate to irreversible cardiac arrest. The death occurred on the same day of her hospitalization. The main diagnosis was dengue; and the cause of death was hemorrhagic shock, with multiple organ failure syndrome. She underwent a complete necropsy the following day, with kidneys exhibiting glomerular and interstitial vascular congestion, erythrophakemia, tubular alterations resulting from hydro-electrolytic disturbance and focal acute tubular necrosis. Dengue NS1 Kit (immunochromatographic test for the detection of the NS1 antigen of dengue virus was used, with a positive result.
- Case 3: Male, aged 10 years, 8 months and 20 days, born in Rio de Janeiro, Brazil, presented fever (38.5 °C) and frontal headache on 14 April 2012 before seeking medical attention in the Emergency Care Unit, since the previous day, he started presenting prostration, non-food vomiting, and with persistent fever. He received initial medical treatment at the emergency care unit. After blood count results on 18 April 2012 (leukopenia, thrombocytopenia), dengue was suspected; then he was referred to the Dengue Center and later for hospitalization. Received venous hydration, fast and maintenance, with hydro-electrolytic control. On 19 April 201, he was acyanotic, with dyspnea, and orotracheal intubation was performed. Severe, shocked, pulseless, required mechanical ventilation. In the same day, serology was performed by using Dengue IgM kit—Elisa Capture (PanBio), with positive result. There was no improvement and having his death recorded on 20 April 2012. The main diagnosis was hemorrhagic dengue; the cause of death was brain edema. At necropsy, the kidneys showed diffuse tubular alterations resulting from hydro-electrolytic disturbance.

The condition of each case is summarized in Table 2 and a more detailed description is contained in the annex present in the Supplementary Materials section.

### 4.4. Histopathological Analysis

The fragments of renal tissue from the autopsy of fatal cases of children were previously fixed in 10% formaldehyde, pH = 7.2, and processed and blocked-in paraffin resin. Sections (5 μm thick) were obtained in glass slides using the microtome (Leica RM-2235). Then, deparaffinized in xylene and rehydrated with decreasing concentrations of ethanol (100 to 70%). Renal tissues were treated hematoxylin and eosin (H.E.) stain and visualized under a light microscope (Olympus BX 53F, Shinjuku, Japan). 400× and 1000× magnifications were used to obtain the images.

### 4.5. Immunohistochemistry Procedure

For immunohistochemical studies, renal tissue sections (4 µm) were deparaffinized in xylol and hydrated in decreasing concentrations of ethanol. The antigenic recovery was performed with citrate buffer (Diagnostic Biosystem, pH 6.0, 96 °C). Then, the peroxidase blocking solution (hydrogen peroxide + 3% methanol) was applied for 10 min. The sections were washed with Tris-HCl (Ph 7.4), and Protein Blocker solution (Spring Bioscience—Code DPB-125) was applied for 10 min. Finally, sections were washed three times in Tris-HCl, and then were incubated overnight at 4 °C with the following primary antibodies: anti-CD56 (1:200 dilution; Vector), anti-CD68 (1:200 dilution; Dako), anti-CD8^+^ (1:500 dilution; DAKO Cytomation, United States), anti-NS3 (1:200 dilution; Expressed in Escherichia coli, purified and inoculated in BALB/c mice), anti-IFN-γ (1:200 dilution; Santa Cruz Biotechnology), anti-TNF-α (1:200 dilution; Abbiotec, United States), anti-RANTES (1:100 dilution; Santa Cruz Biotechnology, United States), anti-VEGFR-2 (1:200 dilution; Spring Bioscience Biotechnology), anti-MMP-9 (1:200 dilution; Santa Cruz Biotechnology), and anti-VCAM-1 (1:200 dilution; Santa Cruz Biotechnology).

On the second day, after the sections remained for 20 min at room temperature, the secondary antibody (REVEAL complement—Spring Bioscience) was applied for 10 min, followed by an anti-mouse or anti-rabbit IgG-HRP conjugate (REVEAL polyvalent HRP—Spring Bioscience) for 15 min. The reactions were revealed with diaminobenzidine (Kit DAB—Diagnostic Biosystems) as a chromogen and then the sections were counterstained with Harris’ hematoxylin (Sigma). Finally, the results were visualized under an optical microscope (Olympus BX 53F, Shinjuku, Japan). 1000× magnification was used to obtain the images. Digital images were rendered using Image Pro Plus software version 4.5.

### 4.6. Kidney Tissue Quantifications/Morphometry

In order to quantify the cell populations, the sections submitted to immunohistochemistry were evaluated using an optical microscope (Olympus BX 53F, Shinjuku, Japan). For each specific antibody stain, 40 images (fields) were acquired randomly (20 from the cortical region and 20 from the medullary region) with a 1000× magnification using the Image Pro version 5.0 software. After capturing the images, positive cells were quantified in each of the 40 fields of the organ and the average positive number of cells was determined. Image acquisition and quantification were performed by an individual blinded to the diagnosis.

The tissue damage was quantified by results of H.E. staining. Forty images of the dengue cases and control were acquired randomly (20 from the cortical region and 20 from the medullary region) with a 1000× magnification using the software Image Pro version 5.0. It used an arbitrary scale of 0–4 (0 = none; 1 = mild; 2 = moderate; 3 = severe; 4 = very severe), implemented according to the degree of the alteration observed in each quadrant of the images. These criteria were adopted according to a previous work that measured the level of organ damage in fatal cases of dengue in adults, based in histopathological alterations [17]. Four criteria were analyzed: Vascular congestion; presence of inflammatory infiltrate; tubular degeneration; and tissue necrosis.

Picrosirius Red staining was performed for visualization under a Nikon ECLIPSE E600 microscope in order to quantify the percentage of collagen area. Forty random images (20 from each region) were captured from slides of dengue cases and control. 400× magnification was used to obtain the images. The areas stained in red/yellow, considered as collagen presence, were quantified by Image-Pro Plus software version 7.0 (Media Cybernetics, Rockville, MD, USA). The result was expressed in the collagen area/total area of the image.

### 4.7. Statistical Analysis

Data were analyzed with the GraphPad Prism software (La Jolla, CA, USA), version 5.02. The number used for both controls and cases was 3 (n = 3). Controls 1–3 and cases 1–3 each represent a single patient. Statistically significant differences between the groups analyzed (controls x cases, each one of them) were determined by the Mann-Whitney test and t-test. The values were considered statistically significant with *p* < 0.05.

## 5. Conclusions

Our findings provide evidence that DENV is able to infect and replicate in the renal tissue, leading to a local inflammatory response, resulting in histopathological changes. It is noteworthy that, in case 1, there was another associated pathogen, which may have contributed even more to a deregulation of the immune response, and consequently to the fatal outcome. In addition, in this work, we indicate possible severity biomarkers (RANTES, VEGFR-2) associated with dengue virus infection in children, aiding in its prognosis and treatment. However, further studies are still necessary to elucidate possible roles for the development of renal injury in dengue cases in children and others viral infections.

## Figures and Tables

**Table 1:** Average serum levels of creatinine and urea of the three children’s fatal cases of dengue during hospitalization days. Creatinine and urea serum levels during hospitalization days, with a reference value for comparison.

	Case 1	Case 2	Case 3	Reference Values
Creatinine (mg/dL)	0.9236	0.6	0.5	0.300–0.800 mg/dL
Urea (mg/dL)	54.54	18.0	19.0	16–42 mg/dL

**Table 2:** Clinical conditions and laboratory tests of each studied case during hospitalization days. The table above provides summarized information about age, symptoms, date of admission to the hospital, laboratory tests made and the date of death of each of the three cases studied.

	Case 1	Case 2	Case 3
Age	7 years and 8 months	9 years, 11 months, 28 days	10 years, 8 months and 20 days
Symptoms	high fever, nausea and vomiting	fever and general malaise; drowsiness and prostration; dehydrated, pale, eupneic, cyanotic, tachycardic, blood pressure of 90 × 50 mmHg, petechiae (face and lower limbs)	frontal headache; presenting prostration, non-food vomiting, and with persistent fever; leukopenia, thrombocytopenia
Admission to Hospital	30 January 2008	24 April 2011	14 April 2012
Laboratory tests	Dengue serology positive Blood culture positive for *Pseudomonas aeruginosa*	Immunochromatographic test for the detection of Dengue virus NS1 antigen: reagent	Dengue serology positive
Death	12 February 2008	25 April 2011	20 April 2012

## Data Availability

All relevant data are within the paper.

## Supplementary Materials

The following supporting information can be downloaded at: https://www.mdpi.com/article/10.3390/pathogens11121543/s1, Table S1: Biochemistry of blood and hemogram of Case 1; Table S2: Biochemistry of blood and hemogram of Case 2; Table S3: Biochemistry of blood and hemogram of Case 3.
