# Supplementary material for: Immunopathology of Renal Tissue in Fatal Cases of Dengue in Children
Source: Pathogens. 2022 Dec 15;11(12):1543. doi: 10.3390/pathogens11121543 (PMC9785549; doi:10.3390/pathogens11121543)
Supplement: Supplementary file 1 [file pathogens-11-01543-s001.zip › case 3 HMJ-121.423-2012-Dengue-2012.pdf]

HMJ-Record Case 3 -Dengue Hemorrhagic

Boy, aged 10 years, 8 months and 20 days, born on 7/30/2001, born in Rio de Janeiro, RJ, presented, at night, fever of 38.5ºC and frontal headache, on 4/14/2012 before seek medical attention at the Emergency Care Unit, as the day before, he had prostration, non-food vomiting, and a fever of 38ºC. He received initial medical treatment at the UPA and was referred to the Dengue Center and then to hospital, after blood count results on 4/18/2012, with leukopenia, thrombocytopenia, suspected of Dengue.

4/18/2012: On physical examination, on admission at 1:30 pm, he was eutrophic (weight 28,500g), hydrated, anicteric, acyanotic, with axillary temperature of 37ºC, respiratory rate of 40irpm, with reduced vesicular murmur in the lung bases, normal cardiac auscultation, blood pressure, lying, 110X62mmHg and sitting, 97x76mmHg, heart rate of 112bpm, filiform peripheral pulses; globular abdomen, painful on superficial and deep palpations. Glascow level of consciousness 15. Strofulous scarring of lower limbs. She received rapid, maintenance venous hydration, with fluid-electrolyte control, and blood was collected for laboratory tests. At 1:30 pm, he was prostrate, tachydyspneic, with an Oxygen Saturation of 94%; abdominal pain and distension, pallor, hydrated (no diuresis); lying blood pressure 104x52mmHg, heart rate 108bpm. Transferred to CTI. Physical examination at the ICU showed drowsiness, swelling, rash, hydrated, ruddy, tachypneic, normal cardiac auscultation, chest auscultation with reduced vesicular murmur in the lung bases, globular abdomen (ascites?) and painful. At 4:30 pm with blood pressure of 105x75mmHg, heart rate of 116bpm and Oxygen Saturation of 96%. **Venous blood tests:** pH 7.242 (N=7.350 to 7.450); PCO2

44.3mmHg (N=35.0 to 45.0); PO2 35.8mmHg (N=80.0 to 100.0); BE -8.5mmol/l, cHCO3 - 18.7mmol/l; SO2 56.8% (N=75.0 to 99.0); K+ 2.54mmol/l (N=3.50 to 4.5); Na+ 146.4mmol/l (N=135.0 to 148.0); Cl 105.3mmol/l (N=98.0 to 107.0); Hb 15.0g/dl (N=11.5 to 17.4); Ht 41.6% (N=35.0 to 50.0); O2Hb (oxyhemoglobin) 54.9% (N=95.0 to 99.0); HHb (deoxyhemoglobin) 41.7% (N=1.0 to 5.0); glucose 204mg/dl (N=60 to 110mg/dl); Lactate 5.8mmol/l (N=0.4 to 2.23).

Blood count on 4/18/2012 (Polo Dengue, CMS Masao Goto)

4/18/2012	1h44min 4h55min 11h29min 4.5 12.3 37.7			normal limits
Millions/mm³ 4.8		83.8 27.3 32.6 13.95.23.6		3.5 to 5.5
Hemoglobin g/dl 12.9		58.1 27.8 14.1 40 14.2		11.5 to 14.5
Hematocrit % 38.1			42.6	35 to 55
VGM page 79.0			79.9	75 to 100
HGM page 26.9			26.7	25 to 35
CHGM g/dl 34.0 Anisocytosis Index RDW % 13.8			33.5	30 to 36
			13.5	11 to 16
Leukocytes Thousand/mm³ 4.5			4.2	3.5 to 10
Granulocytes % 64.9			58.9	35 to 90
Lymphocytes % 21.7			28.2	15 to 50
Undifferentiated leukocytes % 13.4			12.9	2 to 15
Platelets Thousand/mm³	79		23	140 to 440

4/19/2012: Acyanotic, with dyspnea. Orotracheal intubation was performed. Severe, shocked, pulseless, mechanically ventilated (33x8x100%x17), SatO2 96%, hypohydrated; anicteric; acyanotic; absent central and peripheral arterial pulses; isochoric, miotic and photoreactive pupils. Axillary temperature from 36to 35.6 oC; Heart rate from 117 to 121 bpm; Mean Blood Pressure from 90 to 88mmHg; Respiratory rate from 32 to 62 bpm; Diuresis of 3.3ml/kg/h

(oliguria). Auscultation of the chest with abolished breath sounds in the lower 2/3 of the lungs bilaterally, normal cardiac auscultation, heart rate of 106bpm, blood pressure of 76x47mmHg. Distended, painful, ascitic abdomen. Liver 5 cm from the right costal margin. Subjected to conventional treatments for hydroelectrolytic and acid-base control, noradrenaline, etc. At 11 am with anuria. Central pulses present and peripheral pulses absent. Blood pressure of 180x 110mmHg. At 12 pm with diuresis of 0.73ml/kg/h and blood pressure of 140x110mmHg. severe acidosis. Plasma transfusion 10ml/kg. At 12:30 pm with active bleeding from the gastric tube and hematuria. At 16:00, wide pulses, anasarca, blood pressure 120x71mmHg. At 18:30, with heart rate up to 52 bpm, he went into cardiorespiratory arrest; performed external cardiac massage, ventilation with ambu and administered adrenaline, atropine and bicarbonate for 20 minutes, without effectiveness. A bilateral relief thoracic puncture was performed (970ml of pleural fluid was drained). Heart rate return to 156bpm. Blood pressure of 141x76mmHg. SatO2 90%. Median and photoreactive pupils. Right femoral vein puncture was performed and hemodialysis catheter was placed, which started at 6:50 pm. At 20:00, very serious, blood pressure of 65x55mmHg; non-palpable peripheral pulses; weak central pulses; pupils tending to miosis and poorly photoreactive. Good chest expansion. Universally audible vesicular murmur. Normal cardiac auscultation. Ascitic abdomen. At 20:20, blood pressure of 125x85mmHg. At 21:10, blood pressure and 45x40mmHg, heart rate of 100bpm. At 21:20, blood pressure of 65x45mmHg. At 21:50, blood pressure of 47x43mmHg. At 23:20, blood pressure and 90x45mmHg. Blood ultrafiltration (hemodialysis) was started at 11 pm.

4/20/2012: 3h, blood pressure 75x57mmHg, heart rate 150bpm. At 4:10 am, blood pressure 92x46mmHg, heart rate 140bpm.
At 5:50 am, with cardiorespiratory arrest, resuscitation maneuvers and administration of adrenaline, bicarbonate and glucose were performed. At 7 am, **death.** Undergo a complete autopsy.

	4/18/12 venous blood	4/19/12 Central venous blood 9h12min	4/19/12 11:31 am	4/19/12 Venous blood 17h17min	4/19/12 Arterial blood 17h51min	4/19/12 Venous blood 14h54min	4/19/12 arterial blood 9:17 pm	4/19/12 venous blood 9:21 pm
pH	7.242	7.028	6.908	6.794	6.82	6.826	7.293	7.202
PCO_2 mmHG	44.3	74.9	89.3	161.3	137.5	138.5	30.1	34.8
PO_2 mmHg	35.8	70.8	116.5	24.1	105.4	27.9	260.8	31.6
BE mmol/l	-8.5	-12.5	-14.8	-9.7	-10.4	-13.0	-11.2	-
$cHCO^3$ - mmol/l	18.7	19.3	17.4	24.2	21.9	22.4	14.2	13.4
SO2 %	56.8%	81.7	-	35.8	-	38.3	99.3	62.9
K+ mmol/l	2.54	4.71	4.79	6.02	6.44	5.85	2.96	2.20
Na2+ mmol/l	146.4	138.9	142.9	140.6	139.2	138.6	152.4	164.5
Cl -mmol/l	105.3	105.4	105.6	103.2	103.9	104.2	113.8	113.4
Hb g/dl	15.0	12.9	7.7	6.2	4.6	10.1	6.9	3.9
Ht%	41.6	35.3	36.3	25.2	24.0	30.0	17.2	11.4
O2Hb %	54.9	79.2	-	34.0	-	37.1	96.9	60.3
HHb %	41.7	17.7	-	61.1	-	59.6	0.7	35.5
glucose mg/dl	204	135	223	130	110	165	16	-
mmol/l lactate	5.8	4.6	5.7	4.9	4.3	6.0	7.3	-

CBC Limits

HEMOGRAM HMJ

	4/18/2012 1:55 pm	4/18/2012 10:08 pm	4/19/2012 11:08 am	
Leukocytes K/ul	5.66	7.22	15.3	4.60 to 10.2 thousand/microliter
K/ul neutrophils	3.94 (69.5%)	4.96 (68.6%)	10.1 (65.6%)	2.00 to 6.90 K/ul (37.0 to 80.0%N)
K/ul lymphocytes	0.819 (14.5%)	0.930 (12.9%)	1.80 (11.7%)	0.600 to 3.40 K/ul (10.0 to 50.0%L)
Monocytes K/ul	0.800 (14.1%)	0.943 (13.0%)	3.27 (21.3%)	0.00 to 0.900 K/ul (0.00 to 12.0%M)
Eosinophils K/ul	0.013 (0.225%)	0.325 (4.50%)	0.017 (0.111%)	0.00 to 0.700 K/ul (0.00 to 7.00%E)
Basophils K/ul	0.093 (1.64%)	0.70 (0.974%)	0.193 (1.26%)	0.00 to 2.00 K/ul (0.00 to 2.50%B)
RBCs M/ul	5.34	5.58	4.91	4.04 to 6.13 Million/microliter
Hemoglobin g/dl	14.0	16.0	13.4	12.0 to 18.1 g/dl
Hematocrit %	40.4	41.6	37.6	36.0 to 53.7%
VCM fl	75.8	74.6	76.5	MCV: 80.0 to 97.0 femtoliters
HCM pg	26.2	28.6	27.4	HCM: 27.0 to 31.2 picograms
CHCM g/dl	34.5	38.3	35.8	CHCM: 31.8 to 35.4g/dl
Distribution Range of RBCs (RDW) %	10.4	10.5	10.3	RDW: 11.6 to 14.8%
K/ul platelets	40.8	124	22	Platelets: 142,000 to 424,000/microliter
Average Platelet Volume (VPM) fl	-	11.7	9.08	6.80 to 15.0 femtoliters

BIOCHEMISTRY OF BLOOD HMJ

Blood	UV	4/18/2012	4/19/2012	Limits
**Urea**	**method**	15	23	10.0 - 50.0 mg/dl
**creatinine**	jaffe	0.5	0.5	0.700 - 1.300 mg/dl
**Glucose**	Trinder	130	1.3	75.00 - 101.0 mg/dl
**Albumin**	BCG	2.6	2.0	3,500 - 4,800 g/dl
**Globulin (REL)**	REL	1.4	1.3	2,500 - 3,300 (Ratio)
**Total Proteins**	Biuret	4.0	3.3	6,100 - 7,900 g/dl
**TGO / AST**	IFCC	342	1377	0.0 - 38.00 U/l
**TGP / ALT**	IFCC	95	579	0.0 - 41.00 U/l
**GT range**	Modified Szasz	-	94	11.00 - 50.00 U/l
**Alkaline phosphatase**	P-NPP (DGKC)	590	392	85.00 - 300.0 U/l
**Cholesterol**	Trindler	52	-	140.0– 200.0 mg/dl
**triglycerides**	Trindler	98	-	85.00 - 150.00 mg/dl
**Calcium**	Arsenate III	8.3	7.0	8.400 - 10.50 mg/dl
**Phosphor**	UV	-	4.3	4.00 - 7.00 mg/dl
**Magnesium**	Xylidyl Blue	-	1.85	1,900 - 2,500 mg/dl
**PCR**	Turbidimetry	-	14.75	0.0 - 5,000 mg/dl

4/19/2012 SEROLOGY FOR DENGUE [Dengue IgM kit - Elisa Capture (PanBio)]: Reagent

Patient transferred on 4/18/2012 from CMS Masao Goto to Hospital Municipal Jesus with hematocrit of 40% and 40,000 platelets per mm3 , prostrate, tachydyspneic, oliguric, with significant abdominal distension, abdominal pain and satisfactory peripheral perfusion. Transferred from the Intermediate Unit to the Intensive Care Center. The notification of suspected dengue was carried out.

He presented metabolic acidosis, which was performed volumetric expansion with 1000 ml of saline solution. Evolved with improvement in drowsiness and diuresis. At 00:10, hematocrit of 41.6%, after fluid expansion. At 6:40 am on 4/19/2012, he was found to be very uncomfortable, with reduced breath sounds at the lung bases, and was intubated. He was sedated with midazolam, fentanyl and ketamine. It was punctured by the surgeon for central venous access with placement of a 7 Fr double-lumen catheter.

He was evaluated by the nephrologist and continuous hemodialysis was scheduled.

Evolved with dehydration, shock, mixed acidosis, hypotransparency in both hemithoraces.

Administration of norepinephrine, dobutamine and cefepime was started. Two more water expansions were performed with saline solution. An echocardiogram was performed, which was normal. Concentrate of red blood cells, platelets and vitamin K was prescribed. Blood was collected for serology for dengue, blood culture, hemogram and biochemistry. Chest X-ray with the 4 quadrants involved.

Norepinephrine infusion was reduced, because the patient was evolving with a tendency to arterial hypertension. Plasma prescribed. Having a clotting disorder.

At 18:30, the femoral vein was punctured by the surgeon without intercurrences. The radial artery was punctured for better blood pressure control. He remained very serious, in anasarca and acidosis. He evolved with bradycardia, having been performed external cardiac massage, and administration of adrenaline, atropine and bicarbonate. In use of noradrenaline and dobutamine in continuous infusion. Chest drainage was performed bilaterally, with improvement in heart rate, with 970 ml of pleural fluid being drained, with replacement in 2 steps of 500 ml of saline solution.

The left femoral vein was punctured for placement of a hemodialysis catheter by the surgeon, without complications.

He required a progressive increase in noradrenaline infusion. At 9:20 pm, it was associated with continuous infusion of adrenaline.

He had cardiac arrest at 5:50 am, and resuscitation maneuvers were performed with the infusion of an adrenaline bolus, bicarbonate and 25% glucose, without success. Death was confirmed at 7 am on 4/20/2012.

His condition can be classified in ICD 10 under the codes: dengue hemorrhagic fever A91, septic shock A41.9, renal failure N19, respiratory failure T96.9 and clotting disorder D68.9 and anasarca R6O.1.

RIO DE
JANEIRO CITY CITY HALL
Municipal Department of Health and Civil
Defense Undersecretary of Hospital, Urgency and Emergency Care.Jesus
Municipal Hospital
Rua Oito de Dezembro, 717 – Vila Isabel, Rio de Janeiro – RJ CEP 20.550-200Tel.:
2254-0272 ext. 255

PATHOLOGICAL ANATOMY SERVICE

NECROPSY REPORT

Nө :12-802
Date: 4/20/2012

Name: ASF
Registration:121,423/12
Age:10 years old Gender:Male **Date of** birth:30/7/2001

Hospital: Hospital Municipal Jesus
Color: Mulatto
Date of death: 4/20/2017 at 7 am

DIAGNOSES

Cause of Death: Encephalic Edema

MAIN DIAGNOSIS: Hemorrhagic Dengue

(Serology with positive IgM for the arbovirus *Flavivirus sp,* Flaviviridae)

OTHER DIAGNOSES:

1.- Larynx: Chronic laryngitis with foci of erosion and mucosal necrosis, and hemorrhage in the glottic region, associated with reactive lymphoid follicles.
2.- Trachea: Chronic tracheitis with areas of mucosal necrosis and some reactive lymphoid follicles.
3.- Lungs: Bronchitis and bronchiolitis, foci of alveolar atelectasis, anthracosis and foci of pleuritis. Bilateral pulmonary edema.
4.- Heart: Right ventricle and left ventricle with foci of initial ischemic injury in striated muscle fibers. Focal, septal, left subendocardial hemorrhage associated with swelling (hydropic degeneration) of striated muscle fibers.
5.- Submandibular salivary glands: Nonspecific chronic sialoadenitis, in foci, bilateral.
6.- Tongue: Reactive lymphoid follicular hyperplasia.
7.- Esophagus: Mild chronic esophagitis.
8.- Stomach: Chronic mild superficial gastritis, with lymphoid follicles and some foci of necrosis and hemorrhage in the mucosa, and hemorrhage in the submucosa.

9.- Duodenum: Chronic duodenitis with reactive lymphoid follicular hyperplasia and extensive areas of mucosal necrosis and hemorrhage in the mucosa and submucosa.
10.- Jejunum and ileum: Several small foci, in the mucosa, with degeneration and hemorrhage. Nonspecific reactive lymphoid follicular hyperplasia.
11.- Colon and rectum: Nonspecific reactive lymphoid follicular hyperplasia.
12.- Liver: Steatosis. Hemorrhage and necrosis in zones 3 (perivenular) and 2 (mediozonal) of the hepatic lobules, diffusely, associated with passive sinusoidal hyperemia, secondary to acute ischemia due to circulatory failure due to shock; mild to moderate lymphocytic infiltrate in the portal spaces (reactive hepatitis).
13.- Spleen and accessory spleen: Congestion and foci of hemorrhage in the red pulp.
14.- **Lymph nodes:** Secondary lymphocytic depletion, associated with apoptotic lymphocytes and hemogocytosis.
15.- Kidneys: Diffuse tubular alterations resulting from hydroelectrolytic disorders.
16.- Testicles and annexes: Hemorrhage in the spermatic cords, bilaterally.
17.- Neck: Hemorrhage in the soft parts on the right, around the Jugular vein and the Carotid artery, as well as in the soft parts adjacent to the thyroid. Right subclavian hemorrhage.

18.- Bone marrow: General cellularity maintained at the expense of immature and mature granulocytic cells and lymphocytes (some in apoptosis), associated with relative hypoplasia of dysplastic megakaryocytes.
19.- CNS: Hypoxic-ischemic neuronal lesion in cerebral cortex, midbrain, pons, medulla and cerebellum.
20.- Anasarca (generalized edema): Skin and subcutaneous tissue, pulmonary edema, bilateral pleural effusions and moderate ascites.
21.- Ectoscopy: Eutrophic. Right cervical and right subclavian vascular puncture marks, and upper and lower limbs (folds of elbows, wrists, dorsum of hands and feet, and bilateral femoral regions) There are two small, open surgical scars, 1 cm each, bilaterally , on the sides of the chest, 7 cm from the armpits, and old cutaneous scars on the right knee and on theright and left legs.

Fernando Colonna RosmanCRM
52 52023-5
