# Supplementary material for: Immunopathology of Renal Tissue in Fatal Cases of Dengue in Children
Source: Pathogens. 2022 Dec 15;11(12):1543. doi: 10.3390/pathogens11121543 (PMC9785549; doi:10.3390/pathogens11121543)
Supplement: Supplementary file 1 [file pathogens-11-01543-s001.zip › case 1 HMJ-98.030-Dengue-2008.pdf]

HMJ-REPORT Case 1 - HEMORRHAGIC DENGUE

Boy, melanodermic, 7 years and 8 months old, born on 5/10/2000, born in Rio de Janeiro, hospitalized at HMJ on 1/30/2008. On 1/29/2008, after five days of high fever, nausea and vomiting, the mother sought medical care at a health center, being medicated with amoxicillin for diagnosis of "infection."
Local blood count showed: hematocrit: 39.3%; leukocytes: 5,280/µl; red cells: 4.57 x 10 3 /µl; platelets: 70.8 x 10 3 /µl. Not improving, on 1/30/2008, the mother went to the medical assistance unit, where another blood count showed: hematocrit: 45.9%; leukocytes: 8,390/µl; red cells: 5.41 x 10 3 /µl; platelets: 17.9 x 10 3 /µl; the presumptive diagnosis of Dengue is made, and hospital admission is arranged.

Notes: She had chickenpox at age 4. He was born by normal delivery, at term, fraternal twins. She has a mother with tuberculosis, as well as an older brother, out of a total of five siblings. He lives in a brick house, with good sanitary conditions, with running water, without a filter. Lives with chickens and dogs. Next to the residence there is an open sewer.

Physical examination: Marked protein-calorie malnutrition, weighing 15 kg; pale; hypohydrated, acyanotic, anicteric, with slow capillary filling, without petechiae or ecchymosis on the skin. With discreet gingival bleeding. Cardiovascular System: normal cardiac auscultation; low-amplitude arterial pulses; blood pressure of 80x50 mmHg. Respiratory System: pulmonary auscultation with universally audible vesicular murmur, rude, without adventitious sounds; 29 breaths per minute. Abdomen: painful on deep palpation, with sign of peritoneal irritation. Liver 2 cm from the right costal margin. He had moderate hematemesis after abdominal palpation. Child in serious condition, hemodynamically unstable. Admitted to the ICU: received intravenous hydration; platelet concentrate and cardiovascular and respiratory monitoring. Chest radiography showed large pleural effusion
right.

Evolution:

1/31/2008: serious condition; hydrated; pale; afebrile (axillary temperature 36.5°C); tachypneic (36 irpm); 105 heartbeats per minute; blood pressure of 105x61 mmHg; with bilateral periorbital edema; little diuresis (240 ml). Pulmonary auscultation with slightly reduced breath sounds at the right base. He vomited in "coffee grounds" three times.
2/1/2008: Severe general condition, awake, prostrate, pale, acyanotic, icteric, fever of 39.4oC, complaining of abdominal pain and dyspnea. Tachypneic (41 bpm) and tachycardic (130 bpm). Blood pressure of 90x46 mmHg. Pulmonary auscultation with reduced breath sounds in right hemothorax due to pleural effusion, leading to respiratory effort. A small pleural effusion was observed on the left. He underwent right thoracentesis, with the output of 247 ml of citrine yellow pleural fluid, favoring improvement in breathing. Large amount of greenish liquid diarrhea in the morning. Abdomen globose, ascitic, with mild hepatomegaly. Oliguric (diuresis of 96 ml/kg/h). On ventilatory support, with bleeding from the nostrils after aspiration. Anemia and thrombocytopenia.

2/2/2008: In ventilatory prosthesis, sedated, hydrated, afebrile. Hepatosplenomegaly. At night, he did not react to stimuli. Dry mucous membranes, pale, thin pulse. Blood pressure of 81x43 mmHg. Tachycardic (122 bpm). ascites. Anemia and thrombocytopenia. Positive blood culture for *Pseudomonas aeruginosa.*
2/3/2008: Very serious general condition. Afebrile Hemodynamically unstable. Flushed, with periorbital edema in hands and feet. tachycardic. Heart rate ranging from 103 to 149 bpm. Blood pressure ranging from 74x34 mmHg to 112x74 mmHg. Variable respiratory rate from 20 to 39 bpm. Globular abdomen, ascitic. Liver 5 cm from the right costal margin. Spleen not palpable. Right pleural effusion. Oliguric (2.5 ml/kg/h). With anemia and thrombocytopenia.
Epistaxis. Metabolic acidosis.

2/4/2008: Severe, afebrile, in anasarca, pale, acyanotic, anicteric. ascites. Mean arterial pressure [(2PD + PS)/3], ranging from 63 to 94 mmHg. Tachycardic (132 to 179 bpm). Oliguric (1.6 ml/kg/h). With bleeding coming out of the orotracheal tube. He presented bradycardia (38 bpm), being submitted to cardiac massage and intravenous adrenaline.

2/5/2008: Extremely serious, vasodilated, shocked. ascites. Anasarca. Thin pulses, cold extremities, slowed capillary perfusion. Tachycardic (122 to 146 bpm). Mean arterial pressure ranging from 73 to 90 mmHg. Axillary temperature ranging from 35oC to 37.5ºC. Respiratory Distress Syndrome.

2/6/2008: Serious. sedated. hypohydrated. acyanotic. jaundice ascitic. Anasarca. Fine pulses. Axillary temperature: 36 – 37.7ºC. Heart rate from 106 to 147 bpm. Mean arterial pressure: 51 to 93 mmHg. Respiratory rate from 25 to 40irpm. Respiratory Distress Syndrome (chest radiography with diffuse bilateral pulmonary infiltrate and pleuraleffusions). Oliguria (1.4 to 0.33 ml/kg/h). Increased nitrogenous slag. Acute renal failure. Hemodialysis requested. Refractory shock; septic.

2/7/2008: Same as the previous clinical status, with axillary temperature from 35 to 37.5ºC; heart rate from 111 to 145 bpm; mean arterial pressure from 73 to 105 mmHg; respiratory rate of 28 to 30 bpm. On hemodialysis. Refractory shock.

2/8/2008: Same as previous clinical conditions, refractory, septic shock, with axillary temperature of 35 to 37ºC; heart rate from 112 to 159 bpm; mean arterial pressure from 91 to 108 mmHg; respiratory rate of 20 to 32 bpm. On hemodialysis. Echocardiogram: systolic and diastolic myocardial dysfunction, with volume overload. Skin lesions suggestive of vasculitis in the left foot.

2/9/2008: Same as previous clinical states. Axillary temperature of 35ºC; heart rate from 115 to 124 bpm; blood pressure from 111 x 71 mmHg to 125 x 81 mmHg; respiratory rate of 25 to 39 bpm. Skin lesions suggestive of vasculitis on hands and feet. Marked hepatomegaly. On hemodialysis.

2/10/2008: Same as above. Sepsis. SARA. Refractory shock. hemodialysis. Peripheral vasodilation. Vasculitis in digital extremities in hands and feet. Axillary temperature from 36 to 37.6ºC; heart rate from 115 to 130 bpm; mean arterial pressure from 59 to 85 mmHg; respiratory rate from 18 to 30irpm. Chest radiograph with bilateral gross infiltrate. Pulmonary auscultation with coarse vesicular murmur, sub-crepitate rales in bases and diffuse rumbling. Cardiac auscultation with systolic murmur at the left sternal border. Diuresis: 2.5 ml/h. Blood clot exit through the orotracheal tube. Eliminated two ascaris.**2/11/2008:** Same as above. Sepsis. SARA. Refractory shock. hemodialysis. Urinary output: 0.04ml/kg/h. 15ml/day. Axillary temperature: 35ºC to 37ºC. Heart rate: 110 to 120 bpm. Mean blood pressure: 78-110 mmHg. Myocardial dysfunction and volume overload. Respiratory rate: 23 – 52 bpm. Tachypnea. Pleural effusions. Abdominal USG: Moderate ascites. Bleeding from the nostrils.

2/12/2008: Same as above. Sepsis. SARA. Refractory Shock. Blood dyscrasia. Axillary temperature: 35.4 to 38ºC. Heart rate: 107 – 132 bpm. Blood pressure: 151x78 mmHg; 95x53mmHg. Respiratory Rate: 26-72irpm. hemodialysis. Anuria. Orotracheal tube with blood clot outlet. Blood pressure drop. bradycardia. No arterial pulses. Non-responsive to drugs (vasoactive amines). Death at 3:15 pm.

2/13/2008: Submitted to autopsy.

Transfusions:

1/30/2008: Concentrate of platelets and red blood cells.

2/1/2008: Platelet and red blood cell concentrate; plasma (12/12h)

2/2/2008: Concentrated red blood cells

4/2/2008: Plasma and Platelet Concentrate.

2/5/2008: Plasma (12/12h)

2/6/2008: Platelet Concentrate; and plasma (12/12h)

2/7/2008: Plasma 12/12h

8, 9 and 10/2/2008: Concentrate of red blood cells; and plasma (12/12h).

LABORATORY TESTS: BIOCHEMISTRY OF BLOOD (2008)

	1/30	1/2	2/2	3/2	5/2	6/2	8/2	10/2	2/11	2/12	Limits
**Urea**	34.6mg/dl	27.2mg/dl	18.5mg/dl 22.2mg/dl	34.6mg/dl	80.1mg/dl	111mg/dl	17.3mg/dl	63.7mg/dl	40.70mg/dl	95.5mg/dl	10.0 - 50.0
**creatinine**	0.947mg/dl	0.639mg/dl	0.629mg/dl 110mg/	0.947mg/dl	1.24mg/dl	1.74mg/dl 0.331mg/dl	0.992mg/dl	23.8mg/dl	0.531mg/dl	1.24mg/dl	0.300 - 0.800
**Glucose**	dl 87.3mg/dl 111mg/dl	2.80g/dl		110mg/dl	102mg/dl						70.0 - 110
**Albumin**	3.06g/dl	2.64g/dl	3.16g/dl	3.06g/dl					3.77g/dl	3.76mg/dl	3.50 - 4.80
**Total Proteins**	5.26g/dl	4.56g/dl	4.72g/dl 5.15g/dl	5.26g/dl	6.62g/dl					6.81mg/dl	6.10 - 7.90
**TGO**	640U/l	2117U/l	1720U/l	640U/l	291U/l	830U/l		189U/l		146U/l	0.0 - 32.0
**TGP**	175U/l	385U/l	328U/l	175U/l	291.0U/l	137U/l		68.5U/l		59.7U/l	0.0 - 31.0
**GT range**	75.9g/l	85g/l	---	75.9U/l	66.0U/l	73.4U/l		76.5U/l		63.7U/l	
**Phosphatase alkaline**					671U/l	1306U/l		838U/l		476U/l	65 - 645
**bilirubin Total**	2.42mg/dl	---	---	2.42mg/dl	7.39mg/dl	14.6mg/dl		22.8mg/dl		18.6mg/dl	0.0 - 1.00
**bilirubin direct**	1.29mg/dl	---	---	1.29mg/dl	4.85mg/dl	9.88mg/dl		14.8mg/dl		13.5mg/dl	0.0 - 0.20
**Calcium**	7.28mg/dl	7.10mg/dl	7.89mg/dl 7.35mg/dl	7.28mg/dl	8.29mg/dl 7.20mg/dl		9.95mg/dl	9.46mg/dl	10.7mg/dl	9.09mg/dl	8.5 - 10.5
**Magnesium**	1.06mg/dl	---	1.01mg/dl 1.01mg/dl	1.06mg/dl	1.96mg/dl	1.53mg/dl		1.50mg/dl	1.72mg/dl		1.90 - 2.50
**phosphate**	3.64mg/dl	---	1.50mg/dl 2.52mg/dl	3.64mg/dl		5.60mg/dl		0.692mg/dl		2.09mg/dl	4.00 - 7.00
**Sodium**		125meq//	134meq/l	133/135/136meq/l 139meq/l	3.8meq/l		139meq/l	137meq/l	134meq/l	138meq/l	
**Potassium**		4.6meq/l	4.1/5.1/4.4meq/l	3.6meq/l 1.11meq/l			3.7meq/l	4.0meq/l	3.6meq/l	4.7meq/l	
**chlorine**											

HEMOGRAM (2008)

1/29

		1/30	1/30	1/31	1/2	2/2 3/2	4/2		5/2	6/2	7/2	8/2	10/2	2/11	2/12
leukocytes	PAM<br>5.28K/ul	PAM<br>8.39K/ul	6.54K/ul	10.3K/ul	8.11K/ul	15.3K/ul<br>13.6K/ul	13.9K/ul	22.9K/ul	18.3K/ul	25K/ul	29.6K/ul	20.1K/ul	41.1K/ul	26.8K/ul	22.5K/ul
neutrophils	3.95K/ul (74.9%N)	5.58K/ul (6.5%N)	4.05K/ul (61.8%N)	5.28k/ul (51.2%N)	6.80 K/ul (83.9%N)	11.8K/ul (77%N)<br>11.6K/ul (85%N)	10.8K/ul (78.1%N)	18.3K/ul (80.1%N)	15.7K/ul (85.6%N)	20.7K/ul (82.7%N)	26.5K/ul (89.4%N)	18.5K/ul (92.2%N)	38.9K/ul (94.5%N)	25.0K/ul (93.4%N)	21.6K/ul (96.1%N)
lymphocytes	0.838K/ul (15.9%L)	1.57K/ul (18.7%L)	1.21K/ul (18.5%L)	1.52K/ul (14.7%L)	0.558K/ul (6.88%L)	1.81K/ul (11.8%L)<br>1.01K/ul (7.45%L)	1.70K/ul (12.3%L)	2.95K/ul (12.9%L)	1.64K/ul (8.94%L)	1.09K/ul (4.35%L)	1.48K/ul (5.01%L)	1.26K/ul (6.29%L)	0.528K/ul (1.28%L)	0.636K/ul (2.38%L)	0.114K/ul (0.505%L)
monocytes	0.401K/ul (7.60%M)	1.16K/ul (13.9%M)	1.15K/ul (17.6%M)	3.24K/ul (31.4%M)	0.656K/ul (8.10%M)	1.57K/ul (10.3%M)<br>0.839K/ul (6.18%M)	1.19K/ul (8.58%M)	1.54K/ul (6.71%M)	0.856K/ul (4.67%M)	3.12K/ul (12.5%M)	1.53K/ul (5.18%M)	0.263K/ul (1.31%M)	1.65K/ul (4.03%M)	1.03K/ul (3.86%M)	0.719K/ul (3.20%M)
eosinophils	0.014K/ul (0.268%E)	0.021K/ul (0.253%E)	0.018K/ul (0.274%E)	0.008K/ul (0.081%E)	0.005K/ul (0.063%E)	0.011K/ul (0.071%E)<br>0.017K/ul (0.122%E)	0.044K/ul (0.315%E)	0.00 (0.0%E)	0.004K/ul (0.020%E)	0.045K/ul (0.181%E)	0.006K/ul (0.020%E)	0.002K?ul (0.010%E)	0.029K/ul (0.070%E)	0.005K/iu (0.020%E)	0.00 (0.00%E)
Basophiles	0.070K/ul (1.32%B)	0.056K/ul (0.666%B)	0.115K/ul (1.75%B)	0.269 K/ul (2.61%B)	0.82K/ul (1.01%B)	0.124K/ulk (0.810%B)<br>0.134K/ul (0.990%B)	0.106K/ul (0.763%B)	0.073K;ul (0.318%B)	0.144K/ul (0.786%B)	0.66K/ul (0.262%B)	0.123K/ul (0.414%B)	0.035K/ul (0.172%B)	0.029K/ul (0.070%B)	0.103K/ul (0.386%B)	0.037K/ul (0.165%B)
Red Cells	4.57M/ul	5.41M/ul	4.56M/ul	4.82M/ul	3.07M/ul	4.00M/ul<br>3.84M/ul	4.71M/ul	4.10M/ul	4.06M/ul	3.82M/ul	2.95M/ul	2.73M/ul	3.22M/ul	3.63M/ul	3.05M/ul
Hemoglobin	13.3g/dl	16.2g/dl	12.9g/dl	13.9 g/dl	8.60g/dl	11.4g/dl<br>10.6g/dl	12.7g/dl	11.1g/dl	11.2g/dl	11.0g/dl	8.67g/dl	7.76g/dl	8.87g/dl	9.92g/dl	8.23g/dl
hematocrit	39.3%	45.9%	34.5%	35.6%	22.8%	29.7%<br>28.4%	34.9%	30.4%	30.4%	29.1%	22.9%	20.8%	24.4%	27.3%	22.6%
VCM	85.9fl	84.8fl	75.7fl	73.8fl	74.5fl	74.1fl<br>73.9fl	74.2fl	74.2fl	74.8fl	76.2fl	77.8fl	76.2fl	75.8fl	75.2fl	74.2fl
HCM	29.0pg	30.0pg	28.3pg	28.8pg	28.0pg	28.4pg<br>27.5pg	27.0pg	27.1pg	27.7pg	28.7pg	29.4pg	28.4pg	27.5pg	27.3pg	27.0pg
CHCM	33.8g/dl	35.4g/dl	37.4g/dl	39.0g/dl	37.6g/dl	38.3g/dl<br>37.3g/dl	36.4g/dl	36.6g/dl	37.0 g/dl	37.6g/dl	37.8g/dl	37.3g/dl	36.3g/dl	36.4g/dl	36.4g/dl
amplitude of Distribution of Red Cells (RDW)	9.82%	9.54%	7.44%	6.97%	7.47%	7.17%<br>7.37%	8.73%	8.79%	8.77%	9.92%	9.96%	9.87%	9.70%	10.2%	9.94%
platelets	70.8K/ul	17.9K/ul	20.6K/ul	25.7 K/ul	44.2K/ul	28.1K/ul<br>34.4K/ul	38.8K/ul	115K/ul	140K/ul	101K/ul	76.5K/ul	55.4K/ul	27.5K/ul	30.5K/ul	24.9K/ul
platelet (PCT)	0.074%	0.017%	0.023%	0.034%	0.021%	0.020%<br>0.023%	0.024%	0.076%	0.094%	0.67%	0.054%	0.040%	0.022%	0.046%	0.037%
Volume platelet Average (VPM)	10.5fl	9.29fl	11.3fl	13.2fl	4.86fl	7.01fl<br>6.65fl	6.25fl	6.64fl	6.70fl	6.62fl	7.11fl	7.28fl	8.17fl	15.1fl	15.0fl
amplitude of Distribution of platelets (PDW)	19.8 10GSD	22.8 10GSD			16.6 GSD (10GSD)	18.7 (10GSD)<br>17.4 (10GSD)	17.7 (10GSD)	19.4 (10GSD)	18.4 10GSD	18.2 10GSD	18.3 10GSD	18.3 10GSD	20.1 10GSD	-	-

1/29/2008 (PAM) 12h57min: Blood count: leukopenia, lymphopenia, hyperchromic anemia and thrombocytopenia.1/30/2008(PAM)11h33min: Blood count: neutrophilia, lymphopenia, thrombocytopenia.
1/30/2008 HMJ 21:12: CBC: monocytosis, hyperchromic microcytic anemia, thrombocytopenia 1/31/2008 HMJ14:35: CBC: leukocytosis, monocytosis, basophilia, hyperchromic microcytic anemia, thrombocytopenia.
2/1/2008 HMJ 11:51 am and 11:01 pm: CBC: monocytosis, lymphopenia, hyperchromic microcytic anemia, thrombocytopenia, 2/2/2008HMJ 9:50 am and 6:07 pm: CBC: leukocytosis, neutrophilia, monocytosis, hyperchromic microcytic anemia, thrombocytopenia.
2/3/2008 HMJ 2:51 pm: CBC: leukocytosis, neutrophilia, monocytosis, hyperchromic microcytic anemia, thrombocytopenia. 2/4/2008 HMJ 11:32 am: Blood count: leukocytosis, neutrophilia, monocytosis, hyperchromic microcytic anemia, thrombocytopenia.2/5/2008 HMJ 2:43 pm: Blood count: leukocytosis, neutrophilia, monocytosis, hyperchromic microcytic anemia, thrombocytopenia.
2/6/2008 HMJ 10:46 am: Blood count: leukocytosis, neutrophilia, monocytosis, hyperchromic microcytic anemia, thrombocytopenia. ESR: 31mm/h 2/7/2008 HMJ 10:52pm: Blood count: leukocytosis, neutrophilia, monocytosis, hyperchromic microcytic anemia, thrombocytopenia.
8/2/2008 HMJ 16:17: Blood count: leukocytosis, neutrophilia, hyperchromic microcytic anemia, thrombocytopenia.
2/10/2008 HMJ 5:49 pm: Blood count: leukocytosis, neutrophilia, lymphopenia, monocytosis, hyperchromic microcytic anemia, thrombocytopenia.2/11/2008 HMJ 1:37 pm: Blood count: leukocytosis, neutrophilia, monocytosis, hyperchromic microcytic anemia, thrombocytopenia.
2/12/2008 HMJ 12:42 pm: Blood count: leukocytosis, neutrophilia, lymphopenia, hyperchromic microcytic anemia, thrombocytopenia.

HEMOGRAM LIMITS:
Leukocytes: 4.60 to 10.2 K/ul
Neutrophils: 2.00 to 6.90 K/ul (37.0 to 80.0%N)
Lymphocytes: 0.600 to 3.40 K/ul (10.0 to 50.0%L)
Monocytes: 0.00 to 0.900 K/ul (0.00 to 12.0%M)
Eosinophils: 0.00 to 0.700 K/ul (0.00 to 7.00%E)
Basophils: 0.00 to 2.00 K/ul (0.00 to 2.50%B)
Hempacias: 4.04 to 6.13M/ul
Hemoglobin: 12.0 to 18.1 g/dl
Hematocrit: 36.0 to 53.7% VCM: 80.0 to 97.0fl HCM: 27.0 to 31, 2pg CHCM: 31.8 to 35.4g/dl
RDW: 11.6 to 14.8% Platelets: 142,000 to 424,000/ul

CITY HALL OF RIO DE JANEIRO
MUNICIPAL HEALTH SECRETARIAT
MUNICIPAL HOSPITAL JESUS
PATHOLOGICAL ANATOMY SERVICE

NECROPSY REPORT **Nө :** 08-144
Date: 2/13/2008

Name: EV
File no : 98030 **Age:** 7 years and 9 months **Sex:** Male **Color:** Black **Date of birth:** 5/10/2000

Hospitalization date: 1/30/2008
Service: CTI **Death date:** 2/12/2008 at 3:15 pm.

DIAGNOSES

Cause of death: EXTENSIVE BILATERAL PULMONARY HEMORRHAGE

MAIN DIAGNOSIS: HEMORRHAGIC DENGUE

(SEROLOGY WITH POSITIVE IgM FOR DENGUE VIRUS)

OTHER DIAGNOSES:

1. Lungs: Bilateral pneumonitis, with foci of bronchiolitis. Congestion and edema. Extensive bleeding with foci of necrosis. Pleural effusions.
2. Focal chronic tracheitis and laryngitis with areas of mucosal squamous metaplasia. Hemorrhagic content in the lights.
3. Mild focal pericarditis. Pericardial effusion. Myocardial congestion.
4. Tongue with foci of hemorrhage. Secondary hypoplasia of lymphoid tissue.
5. Mild chronic esophagitis and congestion.
6. Superficial mild chronic gastritis in body and pyloric antrum. Congestion.
7. Chronic duodenitis with some lymphoid follicles.
8. Jejunum and ileum with secondary hypoplasia of mucosa-associated and submucosal lymphoid tissue. Ascariasis.
9. Nonspecific chronic colitis and proctitis.
10. Moderate ascites.
11. Nonspecific, focal, chronic sialoadenitis in submandibular salivary glands.
12. Subacute pancreatitis with foci of intralobular edema and acinar necrosis.
13. Hepatomegaly. Acute hepatitis with steatosis, foci of necrosis and centrilobular hemorrhage. Intrahepatic cholestasis. Hemorrhagic shock.
14. Spleen, thymus, lymph nodes and vermiform appendix: Secondary hypoplasia of lymphoid tissue. Congestion. thymic atrophy. splenomegaly.
15. Thyroid: Bleeding into soft tissues adjacent to the thyroid.
16. Pituitary and adrenals: Congestion.
17. Kidneys: Proximal tubular alterations resulting from hydroelectrolytic disturbance. Congestion.
18. Bladder: Congestion of the mucosa.

19. Testicles and epididymis: Congestion. Foci of hemorrhage in the testicular interstitium. Seminiferous tubules, in foci, with psammomas (calcification).
20. CNS: Hypoxic-ischemic lesions mainly in the brainstem and cerebellum. Slight dilation of the lateral cerebral ventricles.
21. Bone marrow: Relative cellular hyperplasia, with a predominance of immature cells, associated with loose aggregates of lymphocytes (many with apoptosis). Megakaryocytic hypoplasia and dysplasia.
22. Pleural fluid (08-161): Autolyzed material. Sparse lymphocytes and squamous epithelial cells. Absence of neoplastic cells.
23. Ectoscopy: Weight loss (protein-caloric malnutrition). Jaundice. Bilateral cervical edema predominantly juxtamastoid. Venipuncture marks on the neck, subclavicular regions and upper and lower limbs. Soft tissue hematoma of the right cervical and subclavian regions. Left heel plantar region with hemorrhage. Dorsum of right wrist with erosion/ulceration and hemorrhage. Free from hypostasis on the back. *rigor mortis.*

Fernando Colonna Rosman
CRM 5252023-5
