# Supplementary material for: Immunopathology of Renal Tissue in Fatal Cases of Dengue in Children
Source: Pathogens. 2022 Dec 15;11(12):1543. doi: 10.3390/pathogens11121543 (PMC9785549; doi:10.3390/pathogens11121543)
Supplement: Supplementary file 1 [file pathogens-11-01543-s001.zip › case 2 HMJ-116.702-2011-Dengue-2011.pdf]

HMJ-Record Case 2-Dengue Hemorrhagic

Girl, 9 years old, 11 months, 28 days old, born on 4/27/2001, born in Itaboraí, RJ, on 4/23/2011 started to have fever (38ºC, 39.5ºC) and general malaise, being taken to the Health Center on 4/24/2011, where a blood count, at 10:27 am, revealed a hematocrit of 36.8% (N=32.7 to 39.6%); Red cells 4.48 million/ mm^3 (N= 4.0 to 4.9 million/mm3), Hemoglobin of 12.9g/dl (N= 10.9 to 13.3g/dl), MCV of 82.1fl (N = 77 to 85fl), HGM 28.8pg, CHGM 35.1%; Leukocytes totaling 2,700/mm^3 (N= 5.0 to 14.5/mm^3) (2% rods with 54/mm^3 (N= 3/mm^3), 75% segmented with 2,025/mm3 (N= 48 to 52 /mm^3), 15% lymphocytes with 405/mm^3 (N=37 to 39/mm^3), 8% monocytes with 216/mm^3 (N=4 to 5/mm^3); Platelets totaling 76,000/mm^3 (N=150,000 to 550,000/mm^3); and Bloodcount, on 4/25/2011, at 5:50 am, showed Hematocrit of 33.4% (N= 37 to 47%) Leukocytes totaling 2,200/mm^3 (N= 4,500 to 10,000/mm^3) and Platelets totaling 22,000 /mm^3 (N=150 mil to 450 mil/mm^3). On physical examination, she presented with drowsiness and prostration, but cooperating with the examination, dehydration, pale, eupneic (respiratory rate of 22irpm, 20irpm, 24irpm), acyanotic, tachycardic (heart rate of 130bpm), blood pressure of 90x60mmHg and 90x50mmHg; axillary temperatures of: 36.2ºC, 38ºC, 37ºC and 36.8ºC. Normal cardiac and chest auscultation. No sign of meningeal irritation. Globular abdomen, with painful hepatomegaly, peristalsis present. Spleen not palpable. Petechiae was observed on the face and lower limbs. Hewas treated with ranitidine, dipyrone, replacement and maintenance hydration with saline and glucose solutions and with the addition of sodium chloride andpotassium chloride. She was transferred to the hospital on 4/25/2011.

Hospital evolution (ICU):

4/25/2011: Spent **10 hours and 40 minutes in the ICU,** receiving hydroelectrolytic replacement and hemodynamic control, with usual medication (albumin, adrenaline, noradrenaline, furosemide, sodium bicarbonate, etc.). At **2 pm,** she was awake, lucid, oriented, pale, with slight swelling of the eyelids, and with tachypnea (O_2 saturation of 96%, with nasal catheter), blood pressure of 79x63 mmHg, 79x50mmHg, heart rate of 128 bpm. At **16:00** she was awake, saying she was fine, with a heart rate of 124 bpm, blood pressure of 88x70x(55)mmHg, respiratory rate of 28-30irpm, diuresis (12:00 to 16:00h) of 565ml. At **18:00** she was awake, with blood pressure of 104x86x63mmHg, heart rate of 160bpm, respiratory rate of 28-32irpm, O2 saturation of 96%. At **18:35, he** hada tachycardia of 180 bpm and a mean blood pressure of 36 mmHg. At **7 pm,** mean arterial pressure was 80mmHg, heart rate was 160bpm, and O2 saturationwas 96%. At **7:35 pm,** heart rate was 160 bpm and blood pressure was unmeasurable (cold shock). From **7:45 pm ,** he presented bradycardia and a drop inblood pressure and a non-palpable peripheral pulse, and external cardiac massage was performed. She was medicated with adrenaline, leading to a tachycardia of 140bpm and a mean blood pressure of 35-40mmHg. Despite efforts to maintain cardiovascular and respiratory functions, through mechanical ventilation, maintenance of blood volume, acid-base balance, with the administration of vasoactive amines, albumin, sodium bicarbonate, etc., he had a meanarterial pressure of 35 mmHg and a fall heart rate from 160bpm to 85bpm, 60bpm, to irreversible cardiac arrest, with death being recorded **at** 10:25 pm . **She underwent a complete necropsy on 4/26/2011.**

Dengue NS1 Kit (immunochromatographic test for the detection of the NS1 antigen of the Dengue virus): Reactive.

4/25/2011
arterial blood
12:15 pm

LABORATORY EXAMS - CTI

		4/25/2011 arterial blood 5:03 pm	Reference values
pH	7.398	7.355	7.350 to 7.450
pCO2 mmHG	28.3	27.4	32.0 to 48.0
pO2 mmHg	73.2	147	83.0 to 108.0
BE mmol/l	-6.8	-9.5	
cHCO3 - mmol/l	17.1	14.9	
SO2 %	96.2	99.7	95.0 to 99.0
K+ mmol/l	4.2	4.5	3.5 to 5.0
Na2+ mmol/l	135	133	136 to 146
Hb g/dl	11.6	12.1	12.0 to 17.5
Ht%	35.7	37.1	
mmol/l lactate	2.0	2.1	0.5 to 1.6

Blood

BIOCHEMISTRY OF BLOOD

	Method 4/25/2011	18.0 0.6 168 2.2		Limits
Urea	UV	1.3 3.5		10.0 - 50.0 mg/dl
creatinine	jaffe	1287		0.700 - 1.300 mg/dl
Glucose	Trinder	453 136		75.00 - 101.0 mg/dl
Albumin	BCG			3,500 - 4,800 g/dl
Globulin (REL)	REL			2,500 - 3,300 (Ratio)
Total Proteins	Biuret			6,100 - 7,900 g/dl
TGO / AST	IFCC			0.0 - 38.00 U/l
TGP / ALT	IFCC			0.0 - 41.00 U/l
GT range	Modified Szasz			11.00 - 50.00 U/l
Alkaline phosphatase	P-NPP (DGKC) -			85.00 - 300.0 U/l
Total Bilirubin	DPD 0.26			0.200 - 1.00 mg/dl
Direct Bilirubin	DPD 0.2			0.100 - 0.300 mg/dl
Indirect Bilirubin	REL 0.1			0.100 - 0.700 mg/dl

BLOOD COUNT

	4/25/2011 12:47 pm	4/25/2011 5:27 pm	CBC Limits
Leukocytes K/ul	2.36	2.97	4.60 to 10.2 mil/microliter
K/ul neutrophils	1.71 (72.6%)	2.08 (70%)	2.00 to 6.90 K/ul (37.0 to 80.0%N)
K/ul lymphocytes	0.267 (11.3%)	0.477 (16.1%)	0.600 to 3.40 K/ul (10.0 to 50.0%L)
Monocytes K/ul	0.353 (15.0%)	0.392 (13.2%)	0.00 to 0.900 K/ul (0.00 to 12.0%M)
Eosinophils K/ul	0.05 (0.214%)	0.004 (0.137%)	0.00 to 0.700 K/ul (0.00 to 7.00%E)
Basophils K/ul	0.022 (0.943%)	0.018 (0.615%)	0.00 to 2.00 K/ul (0.00 to 2.50%B)
RBCs M/ul	4.28	4.73	4.04 to 6.13 Million/microliter
Hemoglobin g/dl	11.7	12.8	12.0 to 18.1 g/dl
Hematocrit %	35.8	39.6	36.0 to 53.7%
VCM fl	83.6	83.7	MCV: 80.0 to 97.0 femtoliters
HCM pg	27.3	27.0	HCM: 27.0 to 31.2 picograms
CHCM g/dl	32.7	32.3	CHCM: 31.8 to 35.4g/dl
Red Blood Cell Distribution Range (RDW) %	7.63	7.70	RDW: 11.6 to 14.8%
K/ul platelets	14.8	23.6	Platelets: 142,000 to 424,000/microliter
Plateletocyte (PCT)	0.019%	0.034%	-
Mean Platelet Volume (MPV) fl	13.0	14.5	

Leukopenia, neutropenia, lymphopenia, anemia, thrombocytopenia.

RIO DE
JANEIRO CITY CITY HALL
Municipal Department of Health and Civil
Defense Undersecretary of Hospital, Urgency and Emergency Care.
Hospital Municipal Jesus Rua
Oito de Dezembro, 717 – Vila Isabel, Rio de Janeiro – RJ CEP 20.550-200 Tel.: 25694088 extension 255

PATHOLOGICAL ANATOMY SERVICE

NECROPSY REPORT

Nº : 11-539
Date: 4/26/2011

Name: MEPDES
Registration: 116,702/11
Age: 9 years **Date of** birth: 4/27/2001
sex: female
Hospital: HMJesus
Color: White **Date of** death: 4/25/2011 at 10:25 pm.

DIAGNOSES

Cause of Death: Hemorrhagic shock. Multiple organ failure syndrome.

MAIN DIAGNOSIS: Dengue (infection by the arbovirus *Flavivirus sp,* Flaviviridae).

(Confirmed by serological test for detection of Dengue virus NS1 Antigen)

OTHER DIAGNOSES: 1. Lungs:
Bronchitis, pneumonitis (foci), congestion, edema, hemorrhage (multifocal), anthracosis, erythrophalkemia.
2. Pleura: Marked bilateral effusion.
3. Larynx: Laryngitis with lymphoid follicles, mucosal edema and congestion.
4. Trachea: Mild tracheitis.
5. Heart: Vascular congestion. Small focus of septal subendocardial hemorrhage in the left ventricle.
6. Submandibular salivary glands: Chronic nonspecific sialoadenitis.
7. Tongue: Lymphoid follicles, from the posterior region, with hemorrhage.
8. Esophagus: Esophagitis with lymphoid follicles. Parietal hemorrhage, focal, in the lower third.
9. Stomach: Vascular congestion, foci of hemorrhage and lymphoid follicles in the mucosa.
10. Duodenum, jejunum, ileum and large intestine: Vascular congestion and lymphoid follicles in the mucosa.
11. Liver: Hepatomegaly. Marked passive hyperemia with hemorrhage and/or necrosis in zones 2 and 3 of the lobules, secondary to acute ischemia (acute circulatory system). Reactive hepatitis. Erythrophakemia.
12. Spleen: Splenomegaly. Erythrophakemia. Red pulp congestion, with foci of extramedullary hematopoiesis and multifocal hemorrhage.

13. Mediastinal and mesenteric lymph nodes: Nonspecific reactive histiocytic hyperplasia. Mild anthracosis in the mediastinum.
14. Vermiform appendix: Reactive follicular hyperplasia.
15. Thymus: Vascular congestion. Hassall's corpuscles with calcification.
16. Bone marrow: Relative cellular hypoplasia with few differentiated cells (predominance of immature cells), associated with megakaryocytic dysplasia. Erythrophakemia.
17. Kidneys: Glomerular and interstitial vascular congestion. Erythrophakemia. Tubular alterations resulting from hydroelectrolytic disturbance. Focal acute tubular necrosis.
18. Bladder: Chronic cystitis with foci of necrosis and hemorrhage in the bladder neck mucosa.
19. Ovaries: Cystic follicles.
20. Peritoneal cavity: Moderate ascites.
21. Anasarca. Pallor. Vascular puncture marks on the upper limbs. Rigor mortis and free of hypostasis on the back.

Fernando C. Rosman
CRM 52 52023-5
